# Patterns of gene expression characterize T1 and T3 clear cell renal cell carcinoma subtypes

**DOI:** 10.1371/journal.pone.0216793

**Published:** 2019-05-31

**Authors:** Agnieszka M. Borys, Michał Seweryn, Tomasz Gołąbek, Łukasz Bełch, Agnieszka Klimkowska, Justyna Totoń-Żurańska, Julita Machlowska, Piotr Chłosta, Krzysztof Okoń, Paweł P. Wołkow

**Affiliations:** 1 Center for Medical Genomics OMICRON, Medical Faculty, Jagiellonian University Medical College, Krakow, Poland; 2 Chair and Department of Urology, Medical Faculty, Jagiellonian University Medical College, Krakow, Poland; 3 Chair of Pathomorphology, Medical Faculty, Jagiellonian University Medical College, Krakow, Poland; Centre National de la Recherche Scientifique, FRANCE

## Abstract

Renal carcinoma is the 20th most common cancer worldwide. Clear cell renal cell carcinoma is the most frequent type of renal cancer. Even in patients diagnosed at an early stage, characteristics of disease progression remain heterogeneous. Up-to-date molecular classifications stratify the ccRCC samples into two clusters. We analyzed gene expression in 23 T1 or T3 ccRCC samples. Unsupervised clustering divided this group into three clusters, two of them contained pure T1 or T3 samples while one contained a mixed group. We defined a group of 36 genes that discriminate the mixed cluster. This gene set could be associated with tumor classification into a higher stage and it contained significant number of genes coding for molecular transporters, channel and transmembrane proteins. External data from TCGA used to test our findings confirmed that the expression levels of those 36 genes varied significantly between T1 and T3 tumors. In conclusion, we found a clustering pattern of gene expression, informative for heterogeneity among T1 and T3 tumors of clear cell renal cell carcinoma.

## Introduction

Renal tumors are classified as the 20th most common malignancy worldwide, both based on incidence and death rates [1]. Clear cell renal cell carcinoma (ccRCC) is the most frequent renal tumor (80–90% of cases)[2,3]. Multiple morphotypes have been described within RCC [4,5] and a growing body of evidence suggests that those morphotypes represent different molecular entities [6–8].

There are several classification systems used to describe renal tumors. Grading is performed by Fuhrman system, based on the nuclear and nucleolar features, and recently modernized by International Society of Urologic Pathology [4]. The most important for prognosis is the stage of the tumor which is evaluated by American Joint Committee on Cancer / The Union for International Cancer Control (AJCC/UICC) TNM system[9]. Although ccRCC cases are usually diagnosed at early stages (in TCGA database, T1 stage represents 48% of all ccRCC cases), clinical outcomes remain heterogeneous within each staging group, suggesting the existence of molecular features unaccounted for by pathology assessment [6,7]. A significant challenge is that metastatic potential and clinical outcome are not well correlated with tumor size and stage [6,7].

In the up-to-date molecular classifications, ccRCC samples are classified into two groups [10]. The authors annotate those clusters ccA and ccB and state that ccA tumors have markedly improved disease-specific survival compared to ccB. Their analyses suggests that the proposed classification was independently associated with survival. However, the heterogeneity within described clusters is significant. An important step in progression of cancer is extension of the tumor beyond the natural limits of the affected organ. In current classification, T1 and T2 tumors differ by their size only, and both are confined to the kidney, while both T3 and T4 tumors extend beyond this organ. Therefore, we decided to select T1 and T3 samples for our study. We aim to verify whether gene expression patterns reflect stage of the disease and to investigate the heterogeneity based on gene expression within the current classification systems in T1 and T3 tumors. Gene expression in ccRCC was studied extensively in the past (exemplary papers:[11–13]). Our study provides additional information on heterogeneity within the samples from various tumor stages as well as points out towards potential mechanisms of transition between these stages.

## Materials and methods

### Sample collection

23 ccRCC tumor samples were collected during radical nephrectomy at the Department of Urology, JUMC. Samples were fixed with formalin and embedded in paraffin at the Department of Pathology for microscopic evaluation and transferred to the Center for Medical Genomics OMICRON for gene expression studies. The study was approved by the Bioethics Committee of the Jagiellonian University.

All patients signed written informed consent forms. Experiments conform to the provisions of the Declaration of Helsinki in 1995 (as revised in Edinburgh 2000). Patient tumors were classified into T1 (13) or T3 (10) stages by a pathologist and independently re-evaluated. Selection of T1 and T3 tumors, as a basis of sample collection for our study, gave prospect to investigate clinically most frequent specimens. In addition, each study group remains homogeneous and sample selection parallels kidney restriction of the tumors in T1 group and extension beyond the kidney in T3 group. Additional clinical data were collected, along with immunohistochemical information summarized in S1 Table.

### RNA isolation

RNA was isolated from 10 x 5 μm slides from Formaldehyde Fixed-Paraffin Embedded (FFPE) block, using Maxwell 16 FFPE Tissue LEV DNA Purification Kit (Promega). Briefly, 300 μl of Mineral Oil and 250 μl of lysis master mix were added per sample and incubated in 56°C for 15 min and subsequently at 80°C for 1 hour. DNA was degraded by DNase I treatment (15 min, RT). The aqueous phase was transferred to Maxwell FFPE Cartridge and RNA was isolated according to Promega RNA—FFPE protocol. 50 μl of Nuclease-Free Water was used for RNA elution. The RNA quantity was measured using NanoDrop 1000 (Thermo Scientific) device and quality was assessed on 2200 TapeStation System (Agilent, RNA ScreenTape), according to manufacturer instructions. DV200 parameter, describing percentage of RNA fragments longer than 200 bases was used for sample classification (S1 Table). Samples with DV200 > 30% were classifies as suitable for further analysis.

### Whole genome DASL assay

The Illumina Whole Genome-DASL assay was performed using 200 ng of RNA following the manufacturer's instructions. Briefly, RNA was reverse transcribed to cDNA using biotinylated primers, followed by immobilization to streptavidin-conjugated paramagnetic particles. Biotinylated cDNAs were then simultaneously annealed to a set of assay-specific oligonucleotides. Extension and ligation of the annealed oligonucleotides generated PCR templates that were amplified using Titanium Taq DNA Polymerase (Clontech). Labeled PCR products were washed and denatured to yield single-stranded fluorescent molecules, which were hybridized to the HumanHT12 v4.0 Whole Genome Gene Expression BeadChips for 16 h at 58°C. The Illumina HiScan was used to scan the arrays.

### Data analysis

Microarray data in *.IDAT format were uploaded and pre-processed in R environment. The ‘beadarray’ package was used to upload the data and ‘lumi’ for normalization and filtration of the data.

### Differential expression analysis

The differentially expressed probes were detected via the Generalized Linear Model framework implemented in the package 'limma'.

For the comparison between T1 and T3 groups as well as the groups defined via hierarchical clustering (A1, A2, A3) the functions 'contrast.fit' and 'eBayes' were used. For analysis of differential expression in the TCGA cohort the framework implemented in the package 'edgeR' was utilized. Gene counts were normalized with the default options and subsequently filtered to reduce the number of hypotheses tested. After estimating the dispersion parameter, the Generalized Linear Model was fitted and tests for coefficients were performed. Since this was used as a replication cohort, we have only recorded the number of genes differentially expressed between the two study groups with the standard level of statistical significance 0.05.

### Hierarchical clustering

The 23 T1 and T3 samples were clustered based on expression of 543 probes. To this aim the function 'hclust' with complete linkage as implemented in the 'heatmap.2' procedure was used. The noticeable pattern where the dendrogram is divided into three main groups was further confirmed with the use of the 'cutreeDynamic' function in the 'dynamicTreeCut' package. The faithfulness of clustering was evaluated using the cophenetic correlation coefficient.

Both the T1 and the T3 samples were clustered based on normalized gene expression values (pseudocounts) generated with 'edgeR' package. To overcome the issue of the Euclidean metric being driven by highly expressed genes, the Renyi divergence function was used as the measure of similarity. Renyi divergence was previously used by the authors of [14] in the context of liver cancer. Once the similarity matrix was estimated, hierarchical clustering was performed as implemented in the function 'hclust'. The optimal number of clusters on each dendrogram, was established via analysis of gap statistics as implemented in the function 'clusGap'.

### Dimension reduction by the t-SNE algorithm

The t-SNE algorithm was used as implemented in R-package 'Rtsne, with all default parameters except for 'perplexity' where 7 was chosen as the value that is expected to produce the least number of 'groupings' for the sample size of 23.

The ROC analysis was based on logistic models with the indicator of the event that the sample is T3 used as the dependent variable. For each of the probes used for hierarchical clustering 300 random training and testing sets were selected (each time the testing set was of size 7) and ROC as well as AUC was calculated as implemented in packages 'ROCR' and “OptimalCutpoints'. Subsequently, for each probe the median AUC was calculated for each sample (taken as the median AUC over all testing sets which contained a given sample). For each sample, the 'goodness' of classification was quantified as the median of these median AUC values over all probes.

### Pathway enrichment

Pathway Enrichment analysis was performed using ‘ClueGO’ plugin for Cytoscape 3.3.0 (http://www.cytoscape.org/, [15]). For all analyses, unless otherwise specified, default Advanced Term/Pathway Selection options were used with Benjamini-Hochberg p-value correction.

## Results

We analyzed 23 ccRCC samples on a microarray platform. Our samples belonged to T1 and T3 stages, as the T2 and T4 stages are rarely diagnosed (only 69 (13%) T2 and 11 (2%) T4 samples in TCGA database). Our main interests were to (1) test the hypothesis if gene expression reflects the histological classification of the JUMC samples (in particular, does the gene expression pattern allow for discrimination between T1 and T3 cases via unsupervised clustering) and (2) whether we will be able to find molecular features that reflect the observed diversity of disease progression.

### Differential gene expression

Differential gene expression comparing T3 vs. T1 samples resulted in 481 genes (543 probes, S2 Table) with adjusted p-value < 0.1 and 181 probes with adjusted p-value < 0.05. The most deregulated genes (36 genes, 41 probes), with |logFoldChange| > 1.5 and adjusted p-value < 0.05, including 2 probes for: GBA3, HAO2, SLC22A2, SLC5A10 (all downregulated) and STEAP3 (upregulated) gives 24 under- and 12 over-expressed genes, presented in Table 1 (heatmap representing those genes is presented in S1 Fig).

**Table 1:** Differentially expressed genes between T3 and T1 groups. Positive and negative FC values correspond to the genes with higher or lower expression in T3 samples, respectively.

Symbol	Gene Name	logFC	P value	adj.P.Val	ENTREZ
ALDOB	aldolase B, fructose-bisphosphate	-3.40	1.20E-05	2.09E-02	229
SLC22A12	solute carrier family 22 (organic anion/urate transporter), member 12	-3.18	1.12E-07	2.99E-03	116085
SLC22A6	solute carrier family 22 (organic anion transporter), member 6	-3.00	1.87E-04	4.61E-02	9356
MIOX	myo-inositol oxygenase	-2.88	5.82E-07	5.01E-03	55586
HAO2	hydroxyacid oxidase 2 (long chain)	-2.51	1.97E-05	2.21E-02	51179
AOC1	amine oxidase, copper containing 1	-2.45	4.66E-05	3.17E-02	26
ANGPTL3	angiopoietin-like 3	-2.35	2.35E-04	4.81E-02	27329
SLC22A2	solute carrier family 22 (organic cation transporter), member 2	-2.32	9.06E-05	3.75E-02	6582
DDC	dopa decarboxylase (aromatic L-amino acid decarboxylase)	-2.27	1.41E-04	4.50E-02	1644
LOC389332	uncharacterized LOC389332	-2.22	2.23E-04	4.81E-02	389332
TRPM3	transient receptor potential cation channel, subfamily M, member 3	-2.20	1.11E-05	2.09E-02	80036
GBA3	glucosidase, beta, acid 3 (gene/pseudogene)	-2.15	1.96E-04	4.61E-02	57733
TMEM27	transmembrane protein 27	-2.11	1.27E-05	2.09E-02	57393
SLC22A2	solute carrier family 22 (organic cation transporter), member 2	-2.09	7.57E-05	3.63E-02	6582
HAO2	hydroxyacid oxidase 2 (long chain)	-2.00	1.04E-05	2.09E-02	51179
SLC5A10	solute carrier family 5 (sodium/sugar cotransporter), member 10	-1.91	2.43E-06	1.39E-02	125206
GBA3	glucosidase, beta, acid 3 (gene/pseudogene)	-1.81	9.36E-05	3.75E-02	57733
NPR3	natriuretic peptide receptor 3	-1.80	8.15E-05	3.68E-02	4883
FLRT3	fibronectin leucine rich transmembrane protein 3	-1.75	8.15E-05	3.68E-02	23767
PAX2	paired box 2	-1.75	9.89E-05	3.92E-02	5076
SLC5A10	solute carrier family 5 (sodium/sugar cotransporter), member 10	-1.67	5.76E-06	1.70E-02	125206
ACE2	angiotensin I converting enzyme 2	-1.65	9.95E-06	2.09E-02	59272
OGDHL	oxoglutarate dehydrogenase-like	-1.65	2.18E-04	4.78E-02	55753
TINAG	tubulointerstitial nephritis antigen	-1.64	4.98E-05	3.17E-02	27283
CDHR2	cadherin-related family member 2	-1.60	1.06E-05	2.09E-02	54825
AQP1	aquaporin 1 (Colton blood group)	-1.52	1.45E-05	2.15E-02	358
FBP1	fructose-1,6-bisphosphatase 1	-1.51	2.25E-04	4.81E-02	2203
MYL3	myosin, light chain 3, alkali; ventricular, skeletal, slow	-1.50	5.02E-05	3.17E-02	4634
ITPKA	inositol-trisphosphate 3-kinase A	1.50	1.49E-04	4.50E-02	3706
EYA1	EYA transcriptional coactivator and phosphatase 1	1.52	2.61E-04	4.97E-02	2138
LOX	lysyl oxidase	1.53	1.73E-04	4.61E-02	4015
STEAP3	STEAP family member 3, metalloreductase	1.56	1.92E-04	4.61E-02	55240
GPRC5A	G protein-coupled receptor, class C, group 5, member A	1.56	1.36E-04	4.46E-02	9052
IGF2BP3	insulin-like growth factor 2 mRNA binding protein 3	1.60	1.62E-05	2.15E-02	10643
TUBB3	tubulin, beta 3 class III	1.65	1.68E-04	4.56E-02	10381
MAP7D2	MAP7 domain containing 2	1.78	1.07E-04	3.94E-02	256714
COMP	cartilage oligomeric matrix protein	1.87	8.99E-06	2.09E-02	1311
STEAP3	STEAP family member 3, metalloreductase	1.90	3.36E-05	2.72E-02	55240
IGFBP1	insulin-like growth factor binding protein 1	1.96	2.16E-04	4.78E-02	3484
TMEM145	transmembrane protein 145	2.07	1.09E-04	3.94E-02	284339

**Fig 1:**
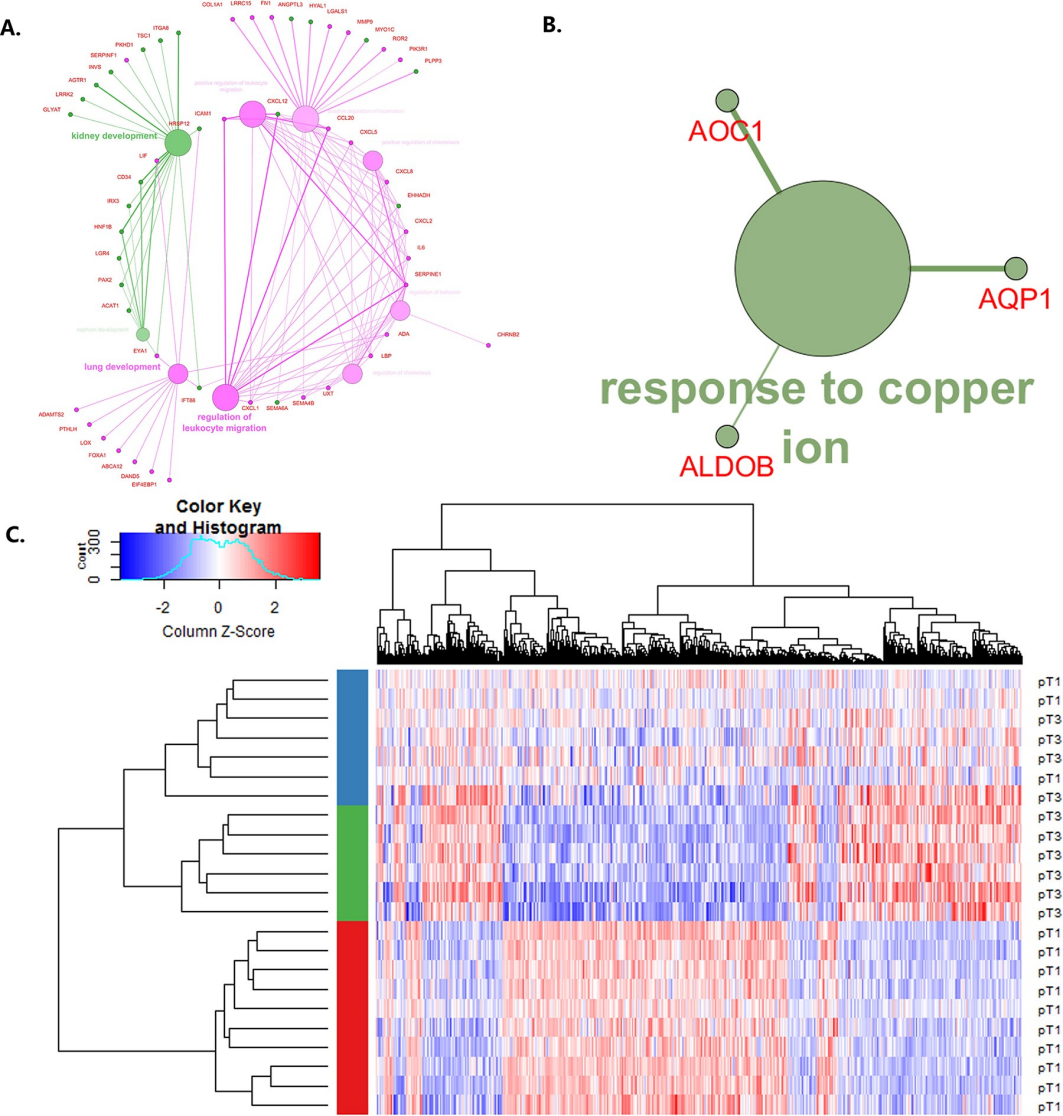
Results of differential expression analysis performed on T3 vs. T1 samples. A. Pathway enrichment comparison performed in ClueGO plugin for Cytoscape software on 481 differentially expressed genes from T3 vs. T1 comparison. Green–downregulated, pink–upregulated genes; the size of the node is inversely proportional to the term p-value. B. Pathway enrichment performed in ClueGO plugin for Cytoscape on gene set with LogFoldChange > |1.5| and adjusted p value < 0.05. C. Heatmap of differentially expressed genes in T3 vs T1 comparison. Based on the expression pattern the samples were divided into three clusters. Color bar indicates what cluster the sample was assigned to: red–A1 (pure T1), green–A3 (pure T3), blue–A2 (mixed).

Pathway Enrichment performed on the differentially expressed gene set (adjusted p-value < 0.1, 481 genes) was narrowed down to those in level 3 in the Genome Ontology (GO) hierarchy. This returned a list of enriched terms, presented in S2 Fig. Further narrowing the results with ‘Use GO Term Fusion’ option reduced the list to 9 GO biological processes terms (Fig 1A) including ‘kidney development’ with corrected p-value 1.31x10-3 (18 associated genes). Interestingly, genes associated with this term were downregulated in T3 samples vs. T1 samples. Analysis of genes with higher log-fold-change values and more stringent adjusted p-value cut-off (0.05) (Table 1) revealed one enriched pathway (Fig 1B)–‘response to copper ion’ with three downregulated genes: aquaporin 1 (Colton blood group, AQP1), amine oxidase, copper containing 1 (AOC1) and aldolase B, Fructose-Bisphosphate (ALDOB).

### Sample clustering

Unsupervised hierarchical clustering, based on expression of 481 genes, divided 23 T1 and T3 samples into three distinct clusters: A1, A2, and A3. Two of the clusters contain populations of T1 (A1) or T3 (A3) samples only, whereas the third cluster includes samples from both groups (A2). This three-cluster pattern (two 'pure' and one mixed) is not present when all (~34K) probes are used for analysis. Therefore, it is unlikely that it is due to a batch effects.

As the distances between the clusters suggests that the A2 cluster is more closely related with A3, despite containing samples from both T1 and T3, we aimed to investigate which expression profiles characterize the A2 group. A heatmap presenting relative gene expressions is shown in Fig 1C.

### Dimension reduction by t-SNE algorithm in the context of sample clustering

To further test whether the pre-selection of features (based on differential expression) allows for faithful sample classification between T1 and T3, an additional machine-learning approach has been adapted. Three sets of probes were used in this analysis: (1) the probes used for hierarchical clustering (aligned to 481 genes); (2) top 40 differentially expressed probes, and (3) all 34476 probes. Subsequently, using these sets of features, samples were projected, using the t-SNE algorithm (see Methods section), on a 3-dimensional space. For the unbiased case (all probes) no association between tumor size and the three components is present. Interestingly, for the two sets of pre-selected features, not only do we see a separation between T1 and T3 samples in the 3D space, but also a separation between the three clusters defined in the previous section. The results are presented in S3 Fig. Additionally, to further test the three-cluster pattern, we applied the UMAP algorithm [16] to project the entire dataset (~33000 probes) onto a 10-dimensional space. Subsequently, we selected three dimensions for which the projection has the strongest association with the clinical diagnosis (T1 vs T3) and visualize the projected data. Interestingly, even in this agnostic approach (with features not being pre-selected) we see a further support for the ‘intermediate cluster’ to appear (see S4 Fig).

### ROC-based classification of T1 and T3 samples in the context of sample clustering

**Fig 2:**
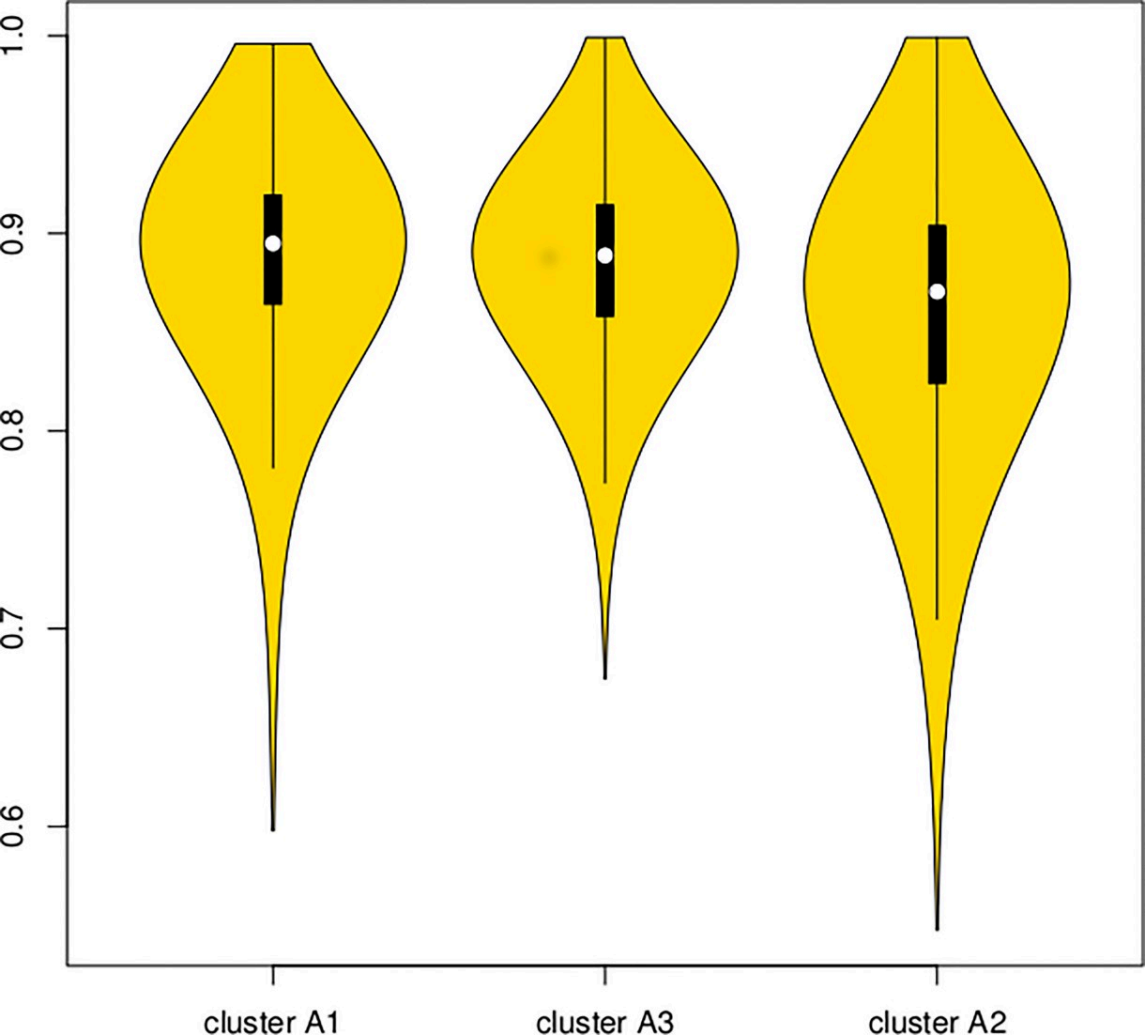
Violin plots of the median AUC based on 300 randomly selected training and testing sets for probes used for hierarchical clustering. The boxplots present median and quartiles. The leftmost violin corresponds to the 'pure T1' cluster, the center corresponds to 'pure T3' and the rightmost to the 'mixed' cluster.

To further test whether there are indeed samples more difficult to correctly classify as T1 or T3 (i.e. samples in the 'mixed cluster'), a ROC-based analysis was performed. For each of the probes aligned to 481 genes, the AUC was calculated for 300 random test subsets of size 7 for a (logistic) model fitted on the remaining 16 training samples. Subsequently, the median for each sample/probe was calculated and the median of these 500 number was assigned to each sample as a measure of 'goodness' of classification. Fig 2 includes violin plots for the 23 samples divided according to the three-cluster pattern. It is clear that the AUC in the 'mixed cluster' is lower than for the two remaining 'pure' clusters.

### Differential variability and clustering faithfulness

In the current study, we use a relaxed threshold (p<0.1) in the process of selection of probes for sample clustering. We wish to support this choice by demonstrating that probes, which are differentially variable between the study groups are more informative about the clustering of samples than the ones with similar variances. To this aim we first compare the variances between T1 and T3 tumors using Levene's test and detect six probes (ILMN_1762410 (SLC22A2), ILMN_1716246 (FRZB), ILMN_1677851 (RARRES1), ILMN_1746128 (ACSM2B), ILMN_3311035 (miR-1251) and ILMN_1793309 (BEND4)) with FDR below the standard 0.05 significance threshold. Secondly, we compare the cophenetic correlation coefficient for two different clusterings: (1) based on differentially expressed probes (p<0.1) with p-value in the Levene's test above the median, and (2) based on differentially expressed probes (p<0.1) with p-value in the Levene's test below the median. We note that for the first set of probes the coefficient equals 0.72 and the second 0.87. Note, that in both of the above clusterings, we use the same number of probes for analysis.

### Characterization of Intermediate Cluster

#### A2 vs A1

First the A2 cluster was compared to A1. In total 13 genes with adj. p-value < 0.05 were found, with the largest log-fold-change = -1.98 achieved by interleukin 6.

#### A2 vs A3

Secondly, A2 and A3 clusters were compared. 22 differentially expressed genes (adj. p-value < 0.05) were identified and the top 15 (with |log-fold-change|>1.5) of them are presented in Table 2. In ClueGO analysis no enriched pathways with at least three genes were found.

**Table 2:** List of differentially expressed genes in A2 vs A3 comparison.

gene symbol	entrez	logFC	P value	adj.P.Val
FKBP9P1	360132	-1.99	5.94E-06	1.09E-02
IGF2BP3	10643	-1.65	4.13E-06	1.02E-02
SLC5A10	125206	1.61	5.67E-06	1.09E-02
NPR3	4883	1.62	1.21E-05	1.89E-02
TMEM171	134285	1.71	1.38E-05	1.98E-02
SLC5A10	125206	1.76	1.81E-06	6.16E-03
GBA3	57733	1.76	1.04E-05	1.71E-02
CYS1	192668	1.80	5.42E-06	1.09E-02
GBA3	57733	2.06	1.96E-05	2.60E-02
TMEM27	57393	2.30	4.80E-07	6.10E-03
PAX2	5076	2.31	5.56E-07	6.10E-03
HAO2	51179	2.33	1.54E-06	6.16E-03
ACSM2B	348158	2.43	6.01E-06	1.09E-02
ACSM2B	348158	2.49	6.76E-06	1.16E-02
SLC22A2	6582	2.59	4.47E-08	1.54E-03
HAO2	51179	2.67	1.76E-06	6.16E-03
SLC22A2	6582	2.85	7.08E-07	6.10E-03
NAT8	9027	2.90	1.66E-06	6.16E-03
AOC1	26	2.99	2.92E-06	8.38E-03
SLC22A2	6582	3.02	1.23E-06	6.16E-03

A list of all differentially expressed probes between A2 and A3 is presented in S3 Table. Main groups/families of genes represented in the results are (trans)membrane proteins, ion-channel proteins or carrier proteins, suggesting a role of regulatory genes and modulation of signal transduction in the observed outcome heterogeneity.

#### A3 vs A1

**Fig 3:**
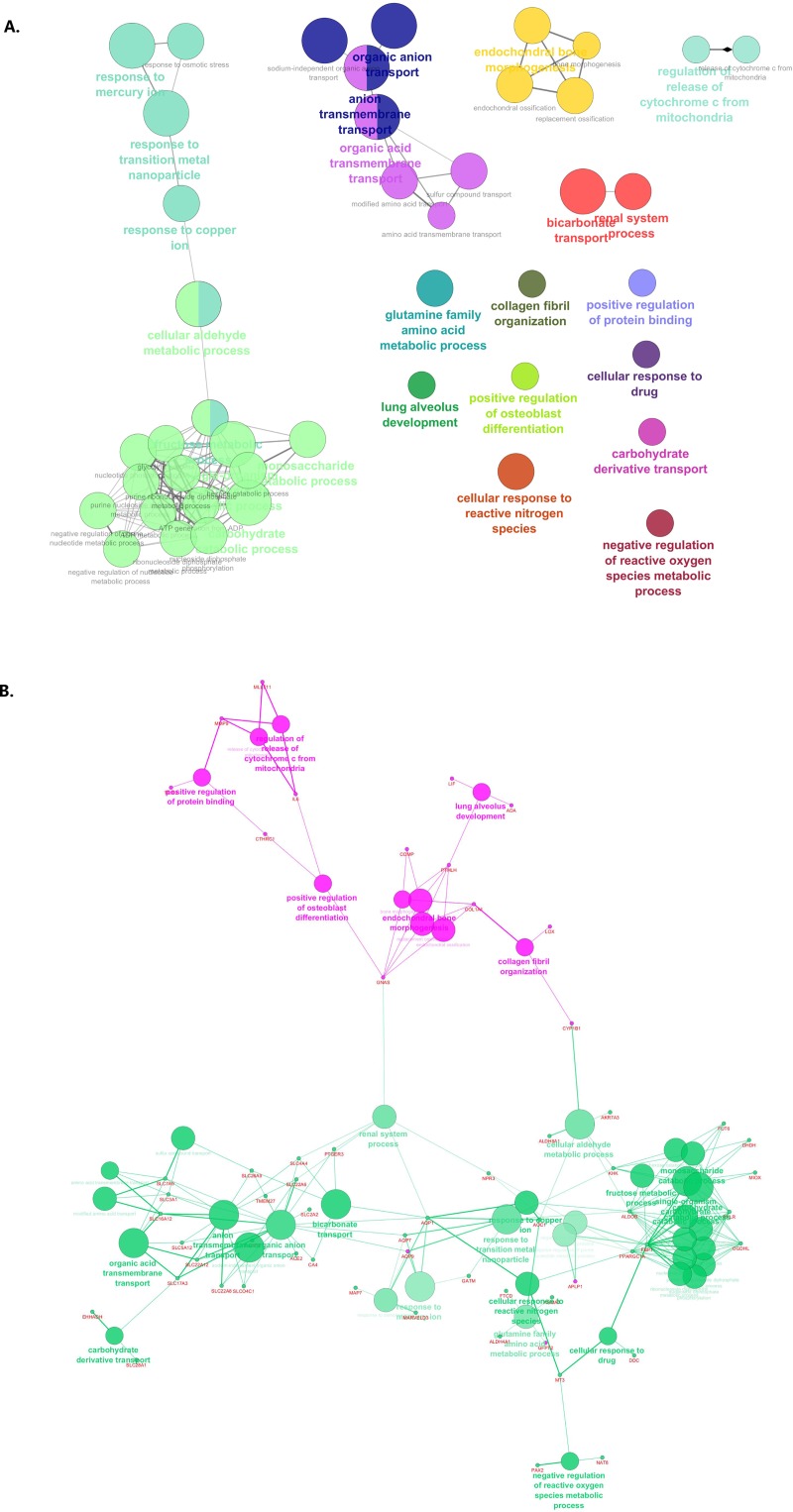
Results of pathway enrichment analysis performed in ClueGO (plugin for Cytoscape software) for A3 vs A1 comparison. A. GO interaction pathway with genes from A3 and A1 initial clusters. B. Indication whether the genes associated with given biological process were up- or down- regulated. Green—GO's associated with down-regulated genes, pink—GO's associated with up-regulated genes.

Differential expression analysis of A3 and A1 clusters revealed a larger set of differentially expressed genes than A1 vs A2 and A3 vs A2. A list of 58 down-regulated and 101 up-regulated probes with |logFoldChange| > |1.5| is presented in S4 Table. ClueGO-based analysis resulted in network depicted in Fig 3A and 3B. Interestingly, genes with lower expression in A3 are associated with morphogenesis and stress response related GO’s and those that are overexpressed with metabolic processes.

#### Validation of results with TCGA data

Our sample size was relatively small, therefore we used TCGA RNA-seq data as a larger replication cohort. Of 481 genes, differentially expressed between T1 and T3 groups, 394 had expression levels available in the TCGA database. Illumina Probe IDs were converted to ENSG# using BioMart. A Gene was considered for further analysis if it was expressed in at least 80% of samples and the median read count exceeded 10.

### T3 vs T1 comparison

**Fig 4:**
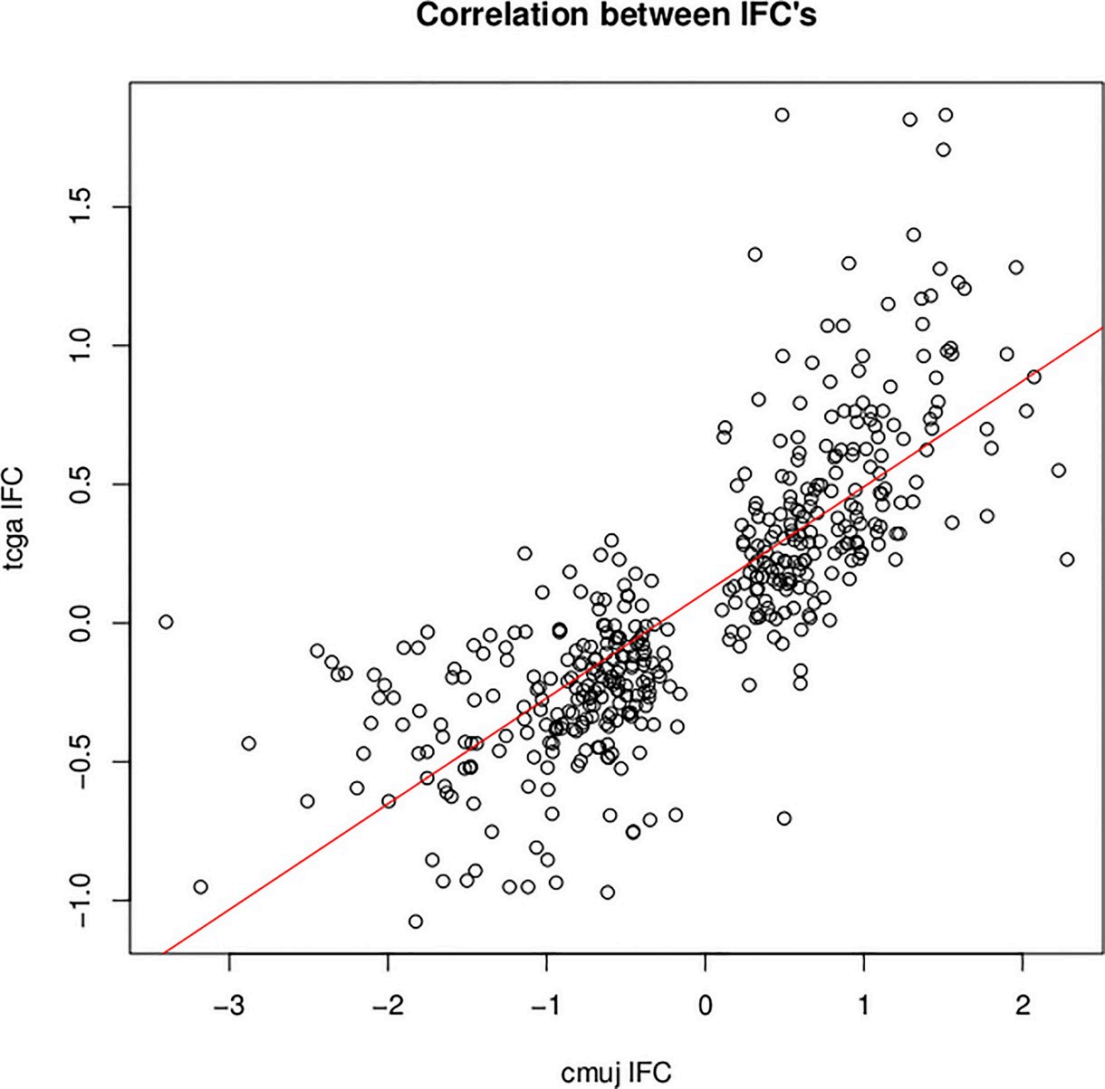
Correlation coefficient plot for the logFoldChanges between the two cohorts–UJ CM and TCGA.

Validation of our primary analysis revealed that almost 67% of differentially expressed genes (264; non-adj. p-value < 0.05) were also differentially expressed in the TCGA RNA-seq data. We additionally note that the correlation coefficient for the logFC’s between the two cohorts equals 0.78, as presented in the Fig 4.

### Hierarchical clustering with Renyi divergence

The TCGA cohort was further used to test the observations made with the use of unsupervised classification. T1 and T3 samples from TCGA were clustered based on each of the 394 differentially expressed genes in the UJ CM cohort. These 394 clusters were then evaluated, using Renyi Divergence measures, for heterogeneity with the expectation that those genes driving the clustering observed in the UJ CM cohort will show evidence of heterogeneity. To this aim, differential expression analysis was performed (between samples in a given cluster versus the largest, reference cluster). The results of this analysis were compared to the set of 36 genes which discriminate between A2 from A1 and A2 from A3.

### T1

Using above-described procedure, T1 samples were divided into 8 clusters (C1-C8), where C1 was the largest and was further used as reference. Results of this analysis are presented in Table 3. Clusters 7 and 8 were excluded from further analysis due to sample size (i.e. the disproportion in the sample size in the case-control design versus the largest cluster).

**Table 3:** Results of differential expression analysis of T1 clusters with sufficient number of samples (Clusters 2 to 6) with cluster 1 (n = 103) as a reference and results of differential expression analysis of T3 clusters with sufficient number of members (Cluster 2 through 4), using Cluster 1 (n = 66) as a reference group.

*Gene Symbol*	*Entrez*	*T1*										*T3*					
		Cluster 2 (n = 91)		Cluster 3 (n = 12)		Cluster 4 (n = 11)		Cluster 5 (n = 42)		Cluster 6 (n = 11)		Cluster 2 (n = 36)		Cluster 3 (n = 43)		Cluster 4 (n = 29)	
		logFC	FDR	logFC	FDR	logFC	FDR	logFC	FDR	logFC	FDR	logFC	FDR	logFC	FDR	logFC	FDR
*A2 vs A1*																	
*KIDINS220*	57498									-0.82	2.10E-05			-0.27	1.53E-02	-0.74	2.94E-07
*MAP7*	9053	0.52	1.36E-05	-0.78	5.00E-03	-0.79	3.56E-03	-1.18	8.05E-14	-2.16	1.37E-12	-0.99	3.68E-05	-1.71	3.26E-15	-2.65	4.03E-16
*IL6*	3569	-0.88	5.45E-03	1.65	3.36E-03	-2.05	7.15E-03	3.17	3.06E-20	3.19	4.27E-11	1.80	9.49E-05	3.57	9.61E-19	4.07	1.89E-18
*ATP6V1G1*	9550					0.97	3.41E-18	-0.20	1.78E-02	-0.55	5.02E-05			-0.24	7.85E-03		
*BACH1*	571	-0.39	2.99E-05			-0.68	7.90E-04							0.47	3.71E-05		
*RCAN1*	1827	0.53	2.13E-03			-1.44	3.20E-04	-0.67	4.80E-03	-1.17	4.15E-03	-0.71	2.06E-02	-1.08	1.79E-05	-1.66	3.49E-06
*PLPP3*	8613					-0.83	6.16E-04	-0.65	7.16E-06	-1.31	3.23E-07			-0.73	1.25E-06	-2.21	5.64E-23
*TRMT10B*	158234							-0.40	2.32E-03	-0.80	3.27E-04			-0.39	1.95E-02		
*FRMD3*	257019			-0.74	8.62E-03	-1.66	6.82E-09	-0.99	5.04E-10	-2.85	4.06E-19	-0.64	2.47E-03	-1.12	2.63E-10	-1.69	3.77E-11
*MYOZ1*	58529			0.97	1.48E-03					-2.29	2.33E-08			0.56	2.37E-02		
*PTAR1*	375743			-0.49	3.95E-02			-0.68	3.08E-07	-1.50	6.71E-10	-0.46	1.32E-03	-0.66	7.06E-08	-0.91	7.45E-08
*RUSC2*	9853					-0.97	3.65E-07	-0.25	3.76E-02	-1.19	2.20E-09	-0.33	4.12E-02	-0.37	4.66E-03		
*SHISA4*	149345	-0.35	1.84E-03	0.97	2.91E-06	0.44	4.96E-02	0.59	2.22E-05	0.92	1.60E-05	0.53	1.12E-02	0.77	4.53E-06	1.33	8.18E-11
*A2 vs A3*																	
*AOC1*	26	0.66	1.90E-02			-4.37	2.76E-08	-0.95	1.40E-02	1.39	6.78E-03						
*ACSM2B*	348158	1.10	5.32E-08	-5.35	1.91E-18	-3.27	3.15E-09	-1.23	1.56E-05	-1.03	3.76E-02			-1.98	1.73E-10	-3.85	7.20E-15
*PAX2*	5076			2.14		-1.62	1.65E-07	-0.77	1.13E-05		3.72E-45			-1.02	1.26E-04	-0.80	2.88E-02
*PTPRH*	5794	-1.06	6.86E-05	-5.98	1.15E-06			3.32	1.70E-30	5.17	4.22E-05	1.49	3.69E-04	2.26	2.10E-10	4.57	6.27E-29
*SLC22A2*	6582			-2.55	1.04E-23	-3.40	1.04E-10	-0.98	2.88E-04	-2.02	1.37E-17			-0.88	1.41E-02	-3.56	9.79E-11
*NPR3*	4883	0.59	1.68E-03	-3.49	4.34E-08	-1.03	1.59E-02	-1.30	2.70E-07	-4.77	5.00E-05	-0.98	2.75E-03	-2.09	1.22E-13	-4.50	5.93E-22
*HAO2*	51179	1.30	1.97E-08	-3.16	3.98E-08	-2.66	1.16E-05	-1.51	3.90E-06	-2.52	4.96E-09	-1.43	3.63E-04	-2.65	2.75E-13	-5.36	2.65E-18
*IGF2BP3*	10643	-1.17	2.29E-04	-5.91	1.85E-04			2.07	1.26E-08	2.92	4.97E-03	2.27	6.80E-07	2.75	2.42E-11	3.66	1.76E-14
*NAT8*	9027	0.93	4.85E-06		4.77E-21	-4.02	3.71E-12	-1.23	1.39E-05	-1.41		-1.05	6.89E-03	-1.65	4.74E-07	-4.03	2.49E-14
*CXCL14*	9547	0.55	4.45E-03	-5.22		-4.03	2.68E-14							-0.92	5.69E-03	-1.00	2.80E-02
*TMEM27*	57393	0.56	5.19E-04	-5.50	2.08E-29	-3.11	1.12E-13	-1.36	2.38E-10	-1.13	2.94E-03	-0.78	3.29E-02	-1.63	3.01E-08	-4.25	1.76E-18
*GLB1L2*	89944					-1.99	2.42E-13										
*SLC5A10*	125206	1.05	1.06E-10	-2.20	9.53E-29	-5.16	6.28E-25	-1.18	1.81E-07	-1.74	2.60E-05	-1.10	9.80E-04	-1.94	2.93E-11	-4.35	1.27E-19
*TMEM171*	134285	0.79	2.02E-07	-2.19	6.53E-09	0.95	1.26E-03	-1.46	2.24E-12			-0.71	3.57E-02	-1.71	3.56E-10	-2.86	5.65E-12
*ALDH1A1*	216			-1.62	2.44E-12	-1.41	2.78E-06			-1.04	5.99E-04			-0.68	2.46E-03	-1.10	2.98E-04
*CLCN5*	1184	0.45	1.10E-04	-0.60	2.16E-09	-0.71	5.97E-03	-1.17	1.71E-14	-1.55	3.13E-08	-0.86	5.32E-06	-1.65	3.33E-22	-2.47	4.46E-22
*ETFDH*	2110	0.41	2.09E-08	-3.24	3.43E-04	0.99	1.23E-12	-0.49	6.63E-07	-0.61	3.52E-04	-0.45	2.97E-05	-0.83	4.72E-18	-1.32	2.34E-22
*TADA2B*	93624					0.66	7.28E-10	-0.28	2.34E-04			-0.25	1.25E-02	-0.25	2.12E-03	-0.35	1.51E-03
*PDZK1*	5174	0.56	1.12E-05	3.32	1.17E-22	-2.79	2.93E-17	-0.74	1.85E-05	-1.02	7.39E-04	-0.82	8.12E-05	-1.63	1.46E-18	-2.72	5.57E-22
*FKBP9P1*	360132	-0.60	2.36E-03	1.44	4.31E-31			1.14	1.35E-06	2.65	4.96E-17	1.72	5.35E-05	2.47	3.20E-11	3.51	5.37E-16
*CYS1*	192668	0.32	4.85E-02	-1.64	1.16E-07			-0.92	9.11E-06	-1.20	1.02E-03	-0.72	3.86E-02	-1.66	2.74E-09	-1.43	2.77E-04
*GBA3*	57733	0.59	2.05E-03		3.15E-04	-2.63	4.64E-08	-1.27	7.61E-07	-2.35	1.29E-06	-1.01	1.18E-03	-2.15	5.05E-15	-5.18	4.29E-28

### T3

T3 samples were also divided into 8 clusters. Clusters 5 to 8 were excluded from further analysis based on sample size. Results of the analysis are presented in Table 3.

## Discussion

Clear cell renal cell carcinoma is the most frequent kidney neoplasm in adults, comprising 80–90% cases of renal tumors [2]. A characteristic feature of ccRCC is large heterogeneity of individual survival times and disease outcomes, even within the same TNM classification groups. Existing pathological classifications do not reflect the molecular basis of the disease [10]. The inability to predict treatment outcome and metastasis in ccRCC could be attributed to the molecular heterogeneity of tumor cells [6,7,17]. Since the high molecular heterogeneity within staging groups could implicitly account for treatment outcome and disease recurrence, we investigated the molecular landscape of ccRCC. We characterized differences in gene expression patterns between T1 and T3 stages in search of genes associated with the molecular heterogeneity of tumors. This approach aimed to identify genes which would be altered between the pure and mixed group (A1 vs A2 and A3 vs A2). The detected genes are involved in regulatory processes and signal transduction. Therefore we hypothesize that the sample heterogeneity can be accounted for by accumulation of subtle deviations in metabolic processes caused by changes in gene expression. We repeated this analysis on the TCGA ccRCC cohort and confirmed 67% of our results. We verified the usability of the gene set to depict the molecular heterogeneity of ccRCC samples.

### Differential gene expression analysis

Among the 36 differentially expressed genes identified between T3 and T1, several have known associations with ccRCC: TRPM3, AQP1, FBP1, ITPKA, LOX, TUBB3, IGFBP1, ALDOB [18–25], other cancer types: FLRT3, ACE2, OGDHL, EYA1, STEAP3, GPRC5A, COMP, [26–32] or other renal diseases: MIOX, TINAG, ANGPTL3 [33–35].

One of the main goals of the study was to emphasize heterogeneity of expression patterns in the context of discrimination between study groups. Therefore, as noted in the Results section (see Differential Variability and Clustering faithfulness) we choose to relax the statistical significance threshold (from 0.05 to 0.1) to include in further analysis genes which have more heterogeneous expression profiles in our cohort and thus higher chance of falling above the standard significance level.

Pathway enrichment analysis emphasized the role of copper metabolism, which is an important process in renal tissue in general, and has a role in cancer development. However, presented genes do not take direct part in pathways regarding those issues.

Other differentially expressed genes include molecular transporters (SLC22A12, SLC22A6, SLC22A2, SLC5A10), (trans)membrane proteins (AOC1, TMEM27, FLRT3, STEAP3, GPRC5A, TMEM145) and other channel proteins (TRPM3, AQP1) involved in regulation and signal transduction in cell metabolism and response to external stimuli. This suggests that dysregulation of signal transduction maybe important in defining the observed diversity of ccRCC outcomes.

### Sample clustering

Clustering of 23 samples, based on all significantly differentially expressed genes, revealed partition of T1 and T3 samples into 3 distinct clusters (Fig 1C). Two of them (A1 and A3) contained only T1 and T3 samples respectively, whereas A2 contained samples from T1 and T3. Interestingly, the gene expression profiles in A2 show no clear pattern of up or downregulation, in contrast to the other two clusters. Therefore we aimed to identify genes involved in molecular heterogeneity–i.e. differentially expressed between A1 vs A2 or A3 vs A2.

Comparison of a A2 with A1 cluster revealed a role for IL-6. Overexpression of IL-6 is associated with enhanced invasiveness and epithelial–mesenchymal transition (EMT) and IL-6 is involved in a JAK/STAT signaling pathway [36]. Although there has been reported lack of correlation between expression of this protein and tumor size or grade [37] our analysis suggests another evidence on regulative role of IL-6 in clear cell renal cell carcinoma.

Comparison of a mixed cluster with pure T3 cluster resulted in 15 genes with |logFoldChange| > 1.5. The 13 overexpressed genes were reported to play a role in RCC: PAX2, NAT8, GBA3, SLC22A2 [38–43] other cancer types: AOC1, HAO2, TMEM27 [44–47], cell death (NPR3 [48]) or kidney metabolism: TMEM171, CYS1 [49,50]. One of the two down-regulated genes–IGF2BP3 is not expressed in normal adult tissues and is known to promote tumor invasion and metastasis [45,51,52]. Some of these same genes were identified as differentially expressed in T3 vs. T1 comparison: HAO2, AOC1, SLC22A2, GBA3, TMEM27, SLC5A10, NPR3, PAX2, IGF2BP3.

The differences shown here lead us to postulate that the isolated intermediate cluster reflects the tumors that are less metastatic prone/aggressive. Several statistically significantly disturbed genes (IL6, GBA3, TMEM27) show contradictory expression change trend to expression changes described in the literature and associated with tumor progression and metastasis [32,38,43].

The 36 genes obtained from A2 vs A1 and A2 vs A3 comparisons code for proteins associated with intracellular signaling and metabolic processes, but lack driver genes or commonly known cancer master regulators, yet these modulators account for the observed sample heterogeneity. This is in line with the previous results of T3 vs T1 comparison and underlies the significance of regulatory/modulatory genes in the progression of the disease.

### Validation of results in TCGA ccRCC cohort

**Fig 5:**
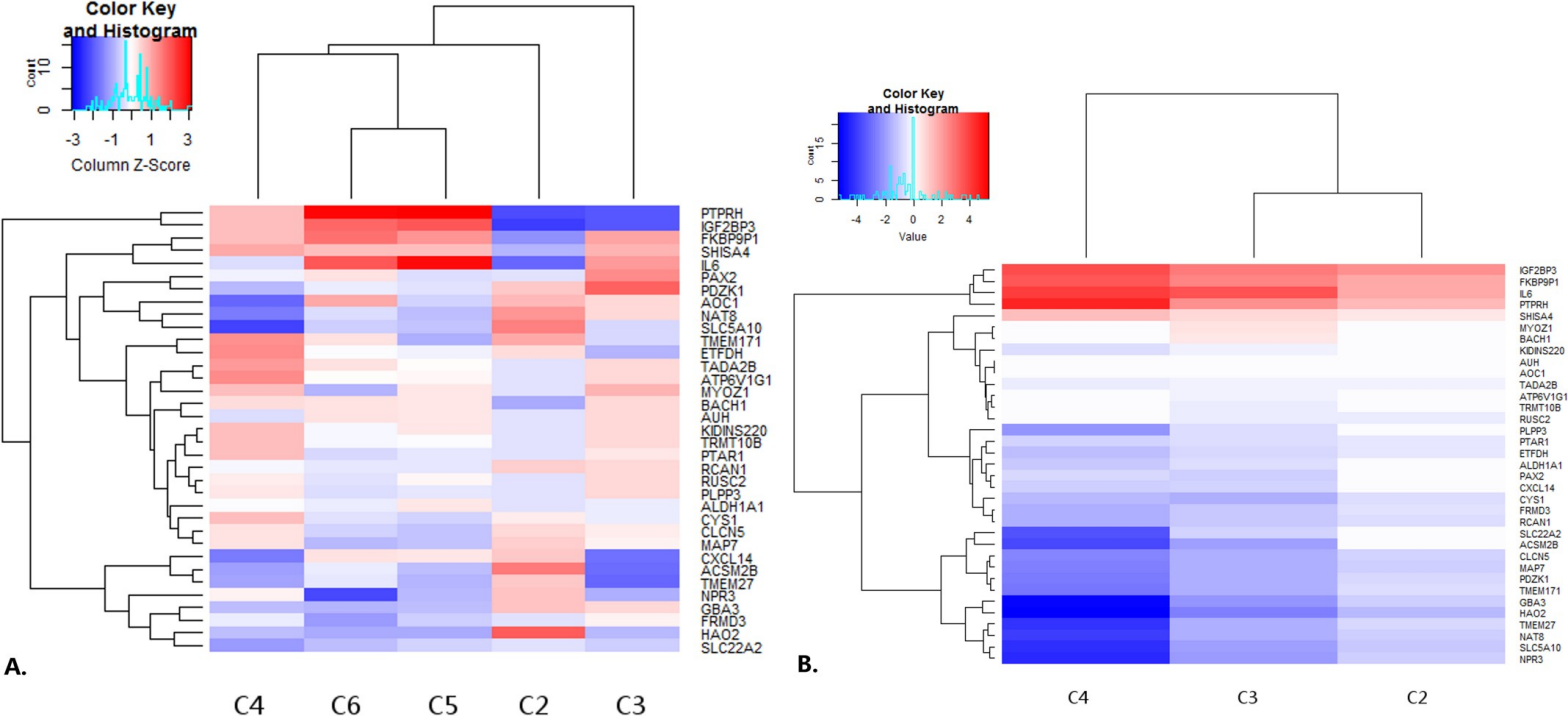
Results of Renyi divergence analysis performed on T1 and T3 data from TCGA database. Gene set resulting from comparisons of three presented clusters were used as an input for clustering method. Next differential expression was calculated in comparison to the largest cluster obtained. Heatmaps show logFoldChange of selected gene set. A. Clusters of T1 samples, B. clusters of T3 samples.

Use of TCGA ccRCC cohort confirmed almost 70% of our results. We tested whether genes differentially expressed between A1/A3 and A2 can be used to measure heterogeneity in a larger dataset. For that purpose, we clustered TCGA ccRCC samples separately T1 and T3 and used the A2-specific genes as an input for differential expression. We found that expression changes in gradual fashion for T3 clusters (Fig 5) suggesting growing dysregulation. Interestingly, the largest T1 clusters (cluster 2 and 5) show contradictory changes in expression suggesting opposite directions of regulatory processes in these samples.

In conclusion we propose that expression of certain RNAs can be used to study the molecular basics of the heterogeneity of ccRCC.

We have found a clustering pattern reflecting heterogeneity of samples. Furthermore, we detected genes associated with diversity of ccRCC samples. We postulate that genes associated with regulatory or signal transduction modulation roles are related to diverse representations of ccRCC occurring regardless of the histological classifications. Further functional research is needed to test these observations.

## Supporting information

S1 Table Clinical parameters of analyzed samples. T, N, M–classification of samples, T_—expanded T classification, diameter–measured in the widest dimension, Grade–ISUP modified Fuhrman grade, survival time–calculated as the number of days between collection date and date of death (calculated when applicable), mdm2 –result of histochemical staining of mdm2 protein, p53—result of histochemical staining of p53 protein, procedure–name of the procedure at which the sample was obtained, necrosis–was the tumor tissue necrotic, DV 200 –Illumina proposed parameter for description of quality of FFPE derived RNA samples (over 30% qualifies sample as sufficient for further analysis).
(DOCX)

S2 Table List of all differentially expressed probes between T3/T1 comparison with adjusted p value under 0.01. ILMN ID–Illumina Probe ID, logFC–logFoldChange of probe expression, AveExpr–average expression of the given probe, P.Value–p value, adj.P.Val–p value adjusted for multiple testing.
(DOCX)

S3 Table List of differentially expressed genes in A2 vs A1 and A2 vs A3 comparisons. All probes that reached adj. p. value < 0.05 cut-off value. ILMN ID–Illumina probe ID, logFC–log Fold Change, AveExpr–average probe expression value, P.Value–p value, adj.P.Val–p value adjusted for multiple testing.
(DOCX)

S4 Table List of differentially expressed genes in A3 vs A1 comparison. All probes that reached adj. p. value < 0.05 and logFC > 1.5 cut-off values. ILMN ID–Illumina probe ID, logFC–log Fold Change, AveExpr–average probe expression value, P.Value–p value, adj.P.Val–p value adjusted for multiple testing.
(DOCX)

S1 Fig Heatmap of differentially expressed genes (24 under- and 12 over-expressed) in T3 vs T1 comparison. Cut-off p-value 0.05. Blue–underexpressed, red–overexpressed genes. Based on the expression pattern the samples were divided into three clusters. Colour bar indicates what cluster the sample was assigned to: red–A1 (pure T1), green–A3 (pure T3), blue–A2 (mixed).
(TIFF)

S2 Fig Pathway Enrichment performed on the differentially expressed gene set. Adjusted p-value < 0.1, 481 genes. Narrowed down to genes in level 3 in the Genome Ontology (GO) hierarchy.
(TIF)

S3 Fig Results of analysis with the tSNE algorithm. Three sets of probes were used in this analysis: (1) the probes used for hierarchical clustering (aligned to 481 genes); (2) top 40 differentially expressed probes, and (3) all 34476 probes. S were projected on a 3-dimensional space. For the unbiased case (all probes) no association between tumor size and the three components is present. Interestingly, for the two sets of pre-selected features, not only do we see a separation between T1 and T3 samples in the 3D space, but also a separation between the three clusters defined in the previous section.
(TIF)

S4 Fig Results of analysis using the UMAP algorithm. Entire dataset (~33000 probes) was projected onto a 10-dimensional space. Three dimensions for which the projection has the strongest association with the clinical diagnosis (T1 vs T3) was selected and projected data visualized. Interestingly, even in this agnostic approach (with features not being pre-selected) we see a further support for the ‘intermediate cluster’ to appear.
(ZIP)

## Abbreviations

AJCC: American Joint Committee on Cancer
ccRCC: clear cell renal cell carcinoma
DiffEx: Differential expression
EMT: epithelial, mesenchymal transition
FFPE: Formaldehyde Fixed-Paraffin Embedded
GO: Genome Ontology
JUMC: Jagiellonian University Medical College
TCGA: The Cancer Genome Atlas
UICC: The Union for International Cancer Control
