# Supplementary material for: Patterns of gene expression characterize T1 and T3 clear cell renal cell carcinoma subtypes
Source: PLoS One. 2019 May 31;14(5):e0216793. doi: 10.1371/journal.pone.0216793 (PMC6544217; doi:10.1371/journal.pone.0216793)
Supplement: S1 Table — T, N, M–classification of samples, T_—expanded T classification, diameter–measured in the widest dimension, Grade–ISUP modified Fuhrman grade, survival time–calculated as the number of days between collection date and date of death (calculated when applicable), mdm2 –result of histochemical staining of mdm2 protein, p53—result of histochemical staining of p53 protein, procedure–name of the procedure at which the sample was obtained, necrosis–was the tumor tissue necrotic, DV 200 –Illumina proposed parameter for description of quality of FFPE derived RNA samples (over 30% qualifies sample as sufficient for further analysis). (DOCX) [file pone.0216793.s001.docx]

**S1 Table** Clinical parameters of analyzed samples. T, N, M – classification of samples, T_ - expanded T classification, diameter – measured in the widest dimension, Grade – ISUP modified

Fuhrman grade, survival time – calculated as the number of days between collection date and date of death (calculated when applicable), mdm2 – result of histochemical staining of mdm2 protein, p53 -

result of histochemical staining of p53 protein, procedure – name of the procedure at which the sample was obtained, necrosis – was the tumor tissue necrotic, DV 200 – Illumina proposed

parameter for description of quality of FFPE derived RNA samples (over 30% qualifies sample as sufficient for further analysis).

| **Necrosis** | yes | no | no | no | no | yes | no | no | no | yes | no | no | no | no | no | yes | yes | no | yes | no | no | yes | yes |
| --- | --- | --- | --- | --- | --- | --- | --- | --- | --- | --- | --- | --- | --- | --- | --- | --- | --- | --- | --- | --- | --- | --- | --- |
| **procedure** | nephrectomy sin | nephrectomy sin | nephrectomy sin | nephrectomy sin | nephrectomy dex | ND | nephrectomy dex | nephrectomy sin | nephrectomy dex | nephrectomy sin | nephrectomy sin | nephrectomy dex | NSS dex, 1 month later nephrectomy dex | nephrectomy dex | nephrectomy sin | nephrectomy dex | nephrectomy dex | nephrectomy dex | nephrectomy sin | nephrectomy dex | nephrectomy dex | ND | ND |
| **p53** | positive | positive | positive | positive | positive | negative | negative | negative | negative | positive | positive | positive | positive | negative | positive | positive | positive | positive | positive | negative | negative | negative | positive |
| **mdm2** | positive | positive | positive | positive | positive | positive | negative | positive | positive | negative | positive | negative | negative | positive | positive | positive | positive | positive | positive | positive | positive | positive | positive |
| **survival time [days]** | 6 | 3209 | 238 |  |  | 70 |  |  |  | 214 |  |  | 4036 | 3042 |  | 764 | 558 | 970 | 1936 | 1806 |  | ND | 1862 |
| **collection date** | 2004-01-16 | 2004-01-29 | 2004-02-12 | 2004-03-17 | 2004-03-25 | 2004-05-17 | 2004-11-09 | 2004-11-22 | 2004-11-24 | 2005-02-01 | 2005-02-02 | 2005-02-14 | 2005-02-25 | 2005-03-15 | 2005-03-16 | 2005-03-16 | 2005-03-16 | 2005-04-05 | 2005-06-27 | 2005-06-27 | 2005-06-29 | 2005-08-04 | 2005-09-05 |
| **date of death** | 2004-01-22 | 2012-11-11 | 2004-10-08 | alive | alive | 2004-07-26 | alive | alive | alive | 2005-09-04 | alive | alive | 2016-03-15 | 2013-07-14 | alive | 2007-04-20 | 2006-09-26 | 2007-12-01 | 2010-10-15 | 2010-06-07 | alive | ND | 2006-10-11 |
| **Delahunt Grade** | 2 | 1 | 1 | 1 | 1 | 4 | 3 | 2 | 2 | 3 | 1 | 1 | 1 | 3 | 1 | 3 | 3 | 1 | 3 | 2 | 1 | 4 | 4 |
| **ISUP Grade** | 2 | 2 | 2 | 1 | 1 | 4 | 4 | 3 | 3 | 3 | 2 | 1 | 1 | 4 | 2 | 3 | 3 | 2 | 3 | 3 | 1 | 4 | 4 |
| **diameter [cm]** | 5 | 3 | 3,5 | 3,5 | 2,2 | 15 | 2,5 | 6 | 4,5 | 6,5 | 5 | 3,5 | 2,2 | 5,5 | 2,8 | 7,5 | 3,5 | 5 | 11,5 | 7 | 5,5 | 11 | 7 |
| **sex** | F | M | M | M | M | M | F | M | M | F | M | F | M | M | F | M | M | F | M | M | F | F | M |
| **age** | 67 | 74 | 79 | 45 | 76 | 73 | 63 | 45 | 67 | 68 | 74 | 69 | 55 | 62 | 72 | 56 | 60 | 52 | 70 | 89 | 66 | 61 | 60 |
| **M** | M0 | M0 | M0 | M0 | M0 | ND | M0 | M1 (liver) | M0 | M0 | M0 | M0 | M0 | M0 | M0 | M1 (liver) | M0 | M0 | M0 | M0 | M0 | ND | ND |
| **N** | N0 | N0 | N0 | N0 | N0 | ND | N0 | N0 | N0 | N0 | N0 | N0 | N0 | N0 | N0 | N1 | N0 | N0 | N0 | ND | N0 | ND | ND |
| **T** | T3 | T1 | T1 | T1 | T1 | T3 | T1 | T1 | T1 | T3 | T3 | T1 | T1 | T3 | T1 | T3 | T1 | T1 | T3 | T3 | T1 | T3 | T3 |
| **T_** | T3b | T1a | T1a | T1a | T1a | T3b | T1a | T1a | T1b | T3b | T3a | T1a | T1a | T3b | T1a | T3b | T1a | T1b | T3b | T3a | T1b | T3b | T3a |
| **A** | A2 | A1 | A1 | A1 | A1 | A2 | A1 | A1 | A2 | A3 | A3 | A1 | A1 | A3 | A1 | A3 | A2 | A1 | A3 | A2 | A2 | A3 | A2 |
| **sample number** | 1529922 | 1530864 | 1531864 | 1534450 | 1535124 | 1538678 | 1550449 | 1551264 | 1551451 | 1555836 | 1556014 | 1556660 | 1557664 | 1558914 | 1558969 | 1558972 | 1558974 | 1560146 | 1565976 | 1566013 | 1566158 | 1568434 | 1570021 |
