# Supplementary material for: Patterns of gene expression characterize T1 and T3 clear cell renal cell carcinoma subtypes
Source: PLoS One. 2019 May 31;14(5):e0216793. doi: 10.1371/journal.pone.0216793 (PMC6544217; doi:10.1371/journal.pone.0216793)
Supplement: S2 Table — ILMN ID–Illumina Probe ID, logFC–logFoldChange of probe expression, AveExpr–average expression of the given probe, P.Value–p value, adj.P.Val–p value adjusted for multiple testing. (DOCX) [file pone.0216793.s002.docx]

**Table 2 SM**. List of all differentially expressed probes between T3/T1 comparison with adjusted p value under 0.01. ILMN ID – Illumina Probe ID, logFC – logFoldChange of probe expression, AveExpr –

average expression of the given probe, P.Value – p value, adj.P.Val – p value adjusted for multiple testing.

| ILMN ID | logFC | AveExpr | t | P.Value | adj.P.Val | B | Gene symbol | Entrez |
| --- | --- | --- | --- | --- | --- | --- | --- | --- |
| ILMN_1747716 | -0,16 | 6,86 | -4,00 | 5,91E-04 | 6,83E-02 | -0,19 | NA | 210 |
| ILMN_1766083 | -0,18 | 6,73 | -4,08 | 4,77E-04 | 6,37E-02 | 0,00 | TMCC3 | 57458 |
| ILMN_2390017 | -0,19 | 6,87 | -4,15 | 4,05E-04 | 6,05E-02 | 0,14 | SOWAHB | 345079 |
| ILMN_1674353 | -0,20 | 6,92 | -3,74 | 1,11E-03 | 8,59E-02 | -0,76 | C2orf91 | 400950 |
| ILMN_1767474 | -0,22 | 12,35 | -4,25 | 3,19E-04 | 5,42E-02 | 0,36 | TXNIP | 10628 |
| ILMN_1731433 | -0,24 | 7,42 | -3,60 | 1,54E-03 | 9,88E-02 | -1,05 | ZNF618 | 114991 |
| ILMN_1695193 | -0,24 | 6,98 | -3,89 | 7,69E-04 | 7,34E-02 | -0,43 | ITFG2 | 55846 |
| ILMN_1762410 | -0,24 | 6,83 | -4,35 | 2,48E-04 | 4,91E-02 | 0,58 | MYO1C | 4641 |
| ILMN_1804283 | -0,24 | 6,92 | -3,80 | 9,43E-04 | 8,11E-02 | -0,61 | LINC01193 | 348120 |
| ILMN_2228463 | -0,25 | 7,46 | -3,86 | 8,22E-04 | 7,59E-02 | -0,49 | ABHD17B | 51104 |
| ILMN_1796316 | -0,26 | 7,55 | -4,76 | 9,00E-05 | 3,76E-02 | 1,49 | PPP2R2D | 55844 |
| ILMN_3239643 | -0,28 | 10,62 | -3,63 | 1,43E-03 | 9,58E-02 | -0,99 | CLTA | 1211 |
| ILMN_1740586 | -0,29 | 8,08 | -3,71 | 1,18E-03 | 8,74E-02 | -0,81 | INVS | 27130 |
| ILMN_2302983 | -0,30 | 9,94 | -3,94 | 6,70E-04 | 7,06E-02 | -0,31 | CNOT6L | 246175 |
| ILMN_2181064 | -0,30 | 7,12 | -3,60 | 1,55E-03 | 9,93E-02 | -1,06 | CCNE1 | 898 |
| ILMN_1772894 | -0,31 | 7,15 | -4,40 | 2,17E-04 | 4,79E-02 | 0,70 | NA | 729428 |
| ILMN_2334193 | -0,32 | 10,24 | -4,07 | 4,93E-04 | 6,46E-02 | -0,03 | NFYC | 4802 |
| ILMN_1789112 | -0,33 | 6,88 | -4,52 | 1,61E-04 | 4,57E-02 | 0,97 | SLC5A10 | 125206 |
| ILMN_1788874 | -0,33 | 7,37 | -3,89 | 7,60E-04 | 7,34E-02 | -0,42 | VPS13A | 23230 |
| ILMN_1663569 | -0,34 | 7,38 | -4,34 | 2,55E-04 | 4,94E-02 | 0,56 | CC2D1B | 200014 |
| ILMN_1702305 | -0,34 | 9,38 | -3,63 | 1,46E-03 | 9,71E-02 | -1,01 | SPG11 | 80208 |
| ILMN_1815480 | -0,35 | 9,58 | -3,62 | 1,47E-03 | 9,72E-02 | -1,01 | ABLIM1 | 3983 |
| ILMN_1777031 | -0,35 | 7,16 | -4,16 | 3,94E-04 | 5,96E-02 | 0,17 | PIP5K1B | 8395 |
| ILMN_1740402 | -0,35 | 9,35 | -4,45 | 1,91E-04 | 4,61E-02 | 0,82 | PURA | 5813 |
| ILMN_1679646 | -0,35 | 7,00 | -3,96 | 6,48E-04 | 6,97E-02 | -0,28 | CACNB2 | 783 |
| ILMN_2387385 | -0,36 | 7,50 | -3,73 | 1,14E-03 | 8,67E-02 | -0,78 | UNC13B | 10497 |
| ILMN_1751232 | -0,37 | 9,43 | -5,97 | 4,84E-06 | 1,67E-02 | 4,08 | TOPORS | 10210 |
| ILMN_2383349 | -0,37 | 8,86 | -3,68 | 1,29E-03 | 9,09E-02 | -0,90 | ACAD10 | 80724 |
| ILMN_1746128 | -0,37 | 7,55 | -3,64 | 1,42E-03 | 9,54E-02 | -0,98 | NUDT6 | 11162 |
| ILMN_1677636 | -0,37 | 9,21 | -3,92 | 7,05E-04 | 7,22E-02 | -0,35 | SLC12A6 | 9990 |
| ILMN_1695157 | -0,37 | 12,27 | -3,61 | 1,53E-03 | 9,85E-02 | -1,05 | SH3GLB2 | 56904 |
| ILMN_1765912 | -0,38 | 8,20 | -3,76 | 1,05E-03 | 8,50E-02 | -0,71 | ZDHHC6 | 64429 |
| ILMN_1664350 | -0,38 | 11,16 | -3,75 | 1,08E-03 | 8,55E-02 | -0,74 | LGR4 | 55366 |
| ILMN_2314169 | -0,38 | 7,13 | -3,84 | 8,58E-04 | 7,73E-02 | -0,53 | PBLD | 64081 |
| ILMN_1665033 | -0,39 | 7,69 | -3,86 | 8,24E-04 | 7,59E-02 | -0,49 | SLC2A11 | 66035 |
| ILMN_2080080 | -0,39 | 11,72 | -4,31 | 2,70E-04 | 5,01E-02 | 0,51 | EIF4EBP2 | 1979 |
| ILMN_1699651 | -0,39 | 10,77 | -3,71 | 1,20E-03 | 8,77E-02 | -0,83 | C11orf1 | 64776 |
| ILMN_1805665 | -0,39 | 6,95 | -3,97 | 6,36E-04 | 6,97E-02 | -0,26 | FAM21C | 253725 |
| ILMN_1715301 | -0,40 | 7,73 | -4,99 | 5,07E-05 | 3,16E-02 | 2,00 | ZFYVE19 | 84936 |
| ILMN_2353490 | -0,40 | 8,30 | -3,64 | 1,42E-03 | 9,54E-02 | -0,98 | AFTPH | 54812 |
| ILMN_1750062 | -0,40 | 12,48 | -3,66 | 1,33E-03 | 9,24E-02 | -0,92 | NDUFB8 | 4714 |
| ILMN_1723678 | -0,40 | 11,26 | -3,91 | 7,25E-04 | 7,32E-02 | -0,38 | PPAP2A | 8611 |
| ILMN_1792744 | -0,40 | 8,41 | -4,57 | 1,42E-04 | 4,49E-02 | 1,08 | GALT | 2592 |
| ILMN_1691503 | -0,41 | 7,26 | -3,95 | 6,63E-04 | 7,04E-02 | -0,30 | ZNF189 | 7743 |
| ILMN_1667018 | -0,42 | 9,93 | -3,97 | 6,28E-04 | 6,97E-02 | -0,25 | LYPLAL1 | 127018 |
| ILMN_1714577 | -0,43 | 7,98 | -3,64 | 1,39E-03 | 9,45E-02 | -0,96 | RUSC2 | 9853 |
| ILMN_1791726 | -0,43 | 7,42 | -3,83 | 8,94E-04 | 7,88E-02 | -0,57 | ISPD | 729920 |
| ILMN_1810684 | -0,43 | 10,64 | -4,13 | 4,25E-04 | 6,13E-02 | 0,10 | MGST2 | 4258 |
| ILMN_2184373 | -0,44 | 7,02 | -4,27 | 2,99E-04 | 5,22E-02 | 0,42 | OR2T3 | 343173 |
| ILMN_1689200 | -0,44 | 12,21 | -4,56 | 1,48E-04 | 4,49E-02 | 1,05 | PKP4 | 8502 |
| ILMN_1718173 | -0,44 | 11,15 | -4,85 | 7,16E-05 | 3,63E-02 | 1,70 | SLC2A4RG | 56731 |
| ILMN_1807423 | -0,44 | 8,12 | -3,97 | 6,36E-04 | 6,97E-02 | -0,26 | IER3IP1 | 51124 |
| ILMN_1690979 | -0,45 | 7,39 | -4,25 | 3,14E-04 | 5,37E-02 | 0,37 | FAM189A2 | 9413 |
| ILMN_1746801 | -0,45 | 9,58 | -4,80 | 8,22E-05 | 3,68E-02 | 1,57 | FBXO3 | 26273 |
| ILMN_1794951 | -0,46 | 7,70 | -3,71 | 1,19E-03 | 8,76E-02 | -0,82 | ASTN2 | 23245 |
| ILMN_2391400 | -0,46 | 7,87 | -3,79 | 9,76E-04 | 8,19E-02 | -0,64 | SNORD116-4 | 6638 |
| ILMN_1682599 | -0,46 | 9,33 | -4,03 | 5,41E-04 | 6,66E-02 | -0,12 | IFT88 | 8100 |
| ILMN_1761577 | -0,46 | 8,91 | -4,42 | 2,09E-04 | 4,74E-02 | 0,74 | TSC1 | 7248 |
| ILMN_1657234 | -0,47 | 10,49 | -3,83 | 8,83E-04 | 7,83E-02 | -0,55 | ATP5A1 | 498 |
| ILMN_1695880 | -0,47 | 10,26 | -4,03 | 5,44E-04 | 6,66E-02 | -0,12 | HABP4 | 22927 |
| ILMN_1734929 | -0,48 | 10,17 | -3,65 | 1,38E-03 | 9,39E-02 | -0,95 | NSMCE4A | 54780 |
| ILMN_1807554 | -0,48 | 6,99 | -4,21 | 3,49E-04 | 5,64E-02 | 0,28 | VIP | 7432 |
| ILMN_1681103 | -0,48 | 7,96 | -3,75 | 1,08E-03 | 8,55E-02 | -0,74 | MYO9A | 4649 |
| ILMN_1728799 | -0,48 | 8,85 | -3,98 | 6,21E-04 | 6,97E-02 | -0,24 | HABP4 | 22927 |
| ILMN_1776516 | -0,49 | 7,82 | -3,96 | 6,39E-04 | 6,97E-02 | -0,26 | MYBBP1A | 10514 |
| ILMN_1798992 | -0,49 | 12,89 | -4,24 | 3,22E-04 | 5,42E-02 | 0,35 | IRX3 | 79191 |
| ILMN_1720034 | -0,50 | 9,67 | -3,84 | 8,70E-04 | 7,81E-02 | -0,54 | ZDHHC14 | 79683 |
| ILMN_1715068 | -0,50 | 9,39 | -4,47 | 1,82E-04 | 4,61E-02 | 0,86 | SIK2 | 23235 |
| ILMN_1749579 | -0,50 | 12,44 | -3,61 | 1,52E-03 | 9,79E-02 | -1,04 | ECHDC2 | 55268 |
| ILMN_2187746 | -0,51 | 9,40 | -4,70 | 1,03E-04 | 3,93E-02 | 1,37 | MTG1 | 92170 |
| ILMN_1678710 | -0,51 | 8,44 | -4,47 | 1,85E-04 | 4,61E-02 | 0,85 | POLR3H | 171568 |
| ILMN_1787815 | -0,51 | 10,60 | -3,72 | 1,17E-03 | 8,74E-02 | -0,81 | ADD3 | 120 |
| ILMN_1740949 | -0,52 | 9,44 | -4,27 | 3,01E-04 | 5,22E-02 | 0,41 | FBXO3 | 26273 |
| ILMN_2091978 | -0,53 | 7,36 | -4,41 | 2,13E-04 | 4,79E-02 | 0,72 | AQP1 | 358 |
| ILMN_2061565 | -0,53 | 8,35 | -3,65 | 1,37E-03 | 9,39E-02 | -0,95 | RBPMS | 11030 |
| ILMN_1763834 | -0,53 | 11,43 | -3,62 | 1,48E-03 | 9,73E-02 | -1,02 | CDS1 | 1040 |
| ILMN_1723443 | -0,54 | 10,13 | -4,37 | 2,34E-04 | 4,81E-02 | 0,63 | TSC1 | 7248 |
| ILMN_1685616 | -0,54 | 7,83 | -4,24 | 3,24E-04 | 5,42E-02 | 0,35 | LRRC48 | 83450 |
| ILMN_2199389 | -0,54 | 9,04 | -4,01 | 5,73E-04 | 6,76E-02 | -0,17 | CPT1A | 1374 |
| ILMN_1770725 | -0,54 | 8,29 | -3,79 | 9,81E-04 | 8,19E-02 | -0,65 | KANK1 | 23189 |
| ILMN_1680339 | -0,55 | 7,70 | -3,79 | 9,78E-04 | 8,19E-02 | -0,65 | C11orf54 | 28970 |
| ILMN_1725090 | -0,55 | 9,38 | -3,93 | 6,88E-04 | 7,12E-02 | -0,33 | IVNS1ABP | 10625 |
| ILMN_2378654 | -0,55 | 12,29 | -4,44 | 1,99E-04 | 4,62E-02 | 0,78 | HDHD3 | 81932 |
| ILMN_1806710 | -0,55 | 11,99 | -5,23 | 2,86E-05 | 2,69E-02 | 2,52 | FBXL5 | 26234 |
| ILMN_1711748 | -0,55 | 10,76 | -3,88 | 7,94E-04 | 7,44E-02 | -0,46 | ZBTB46 | 140685 |
| ILMN_1743620 | -0,56 | 7,02 | -4,43 | 2,01E-04 | 4,62E-02 | 0,77 | SLC22A13 | 9390 |
| ILMN_1693338 | -0,56 | 12,32 | -3,75 | 1,08E-03 | 8,55E-02 | -0,74 | OPLAH | 26873 |
| ILMN_1709674 | -0,56 | 7,63 | -3,99 | 5,99E-04 | 6,89E-02 | -0,21 | FANCC | 2176 |
| ILMN_1713246 | -0,56 | 9,10 | -4,84 | 7,32E-05 | 3,63E-02 | 1,68 | SURF1 | 6834 |
| ILMN_1676822 | -0,57 | 7,37 | -4,86 | 6,94E-05 | 3,57E-02 | 1,72 | DHTKD1 | 55526 |
| ILMN_1761275 | -0,57 | 9,11 | -3,70 | 1,22E-03 | 8,83E-02 | -0,85 | HNF1B | 6928 |
| ILMN_2085844 | -0,57 | 7,28 | -5,42 | 1,80E-05 | 2,20E-02 | 2,93 | LGALS2 | 3957 |
| ILMN_1746013 | -0,58 | 11,05 | -4,53 | 1,56E-04 | 4,57E-02 | 1,00 | CLYBL | 171425 |
| ILMN_1738725 | -0,58 | 9,80 | -3,76 | 1,04E-03 | 8,47E-02 | -0,70 | SIRT5 | 23408 |
| ILMN_2223313 | -0,58 | 8,88 | -3,62 | 1,49E-03 | 9,74E-02 | -1,03 | EFCAB6 | 64800 |
| ILMN_1772131 | -0,59 | 7,57 | -3,71 | 1,19E-03 | 8,76E-02 | -0,82 | ZNF677 | 342926 |
| ILMN_1695579 | -0,59 | 7,14 | -4,95 | 5,56E-05 | 3,28E-02 | 1,92 | LRRC66 | 339977 |
| ILMN_1752214 | -0,59 | 7,93 | -3,81 | 9,32E-04 | 8,07E-02 | -0,60 | DDAH1 | 23576 |
| ILMN_1735445 | -0,59 | 7,66 | -3,64 | 1,42E-03 | 9,54E-02 | -0,98 | ALDH3A2 | 224 |
| ILMN_1728009 | -0,60 | 8,06 | -3,87 | 8,01E-04 | 7,48E-02 | -0,47 | FBXO15 | 201456 |
| ILMN_1729180 | -0,60 | 7,77 | -3,66 | 1,34E-03 | 9,24E-02 | -0,93 | EMCN | 51705 |
| ILMN_1803686 | -0,60 | 8,29 | -3,98 | 6,10E-04 | 6,96E-02 | -0,22 | LYRM9 | 201229 |
| ILMN_2408748 | -0,61 | 9,83 | -3,84 | 8,59E-04 | 7,73E-02 | -0,53 | PLCL2 | 23228 |
| ILMN_1744381 | -0,61 | 7,65 | -4,38 | 2,31E-04 | 4,81E-02 | 0,65 | SOX6 | 55553 |
| ILMN_1790761 | -0,61 | 7,66 | -4,08 | 4,74E-04 | 6,37E-02 | 0,00 | KCNJ15 | 3772 |
| ILMN_2196328 | -0,62 | 9,55 | -4,15 | 4,03E-04 | 6,04E-02 | 0,15 | MPC1 | 51660 |
| ILMN_1668510 | -0,62 | 7,25 | -3,82 | 9,12E-04 | 7,99E-02 | -0,58 | AGTR1 | 185 |
| ILMN_2412192 | -0,62 | 7,10 | -4,69 | 1,06E-04 | 3,93E-02 | 1,35 | NA | 283887 |
| ILMN_1668865 | -0,62 | 10,53 | -5,52 | 1,39E-05 | 2,15E-02 | 3,15 | HINT2 | 84681 |
| ILMN_1700728 | -0,62 | 7,54 | -3,68 | 1,28E-03 | 9,05E-02 | -0,89 | GLYAT | 10249 |
| ILMN_2193553 | -0,62 | 8,48 | -4,05 | 5,13E-04 | 6,52E-02 | -0,07 | PARM1 | 25849 |
| ILMN_2230178 | -0,62 | 7,80 | -3,59 | 1,57E-03 | 1,00E-01 | -1,07 | DMGDH | 29958 |
| ILMN_1731503 | -0,62 | 8,42 | -4,18 | 3,71E-04 | 5,71E-02 | 0,22 | ARSD | 414 |
| ILMN_2301083 | -0,63 | 10,38 | -3,80 | 9,61E-04 | 8,16E-02 | -0,63 | PPAP2A | 8611 |
| ILMN_2197381 | -0,63 | 8,47 | -4,13 | 4,22E-04 | 6,13E-02 | 0,11 | NA | 6928 |
| ILMN_1803197 | -0,63 | 9,98 | -3,62 | 1,48E-03 | 9,73E-02 | -1,02 | SYNE1 | 23345 |
| ILMN_1740076 | -0,64 | 8,59 | -3,73 | 1,12E-03 | 8,63E-02 | -0,77 | C19orf18 | 147685 |
| ILMN_1768506 | -0,64 | 8,99 | -3,75 | 1,08E-03 | 8,55E-02 | -0,74 | PLEKHA6 | 22874 |
| ILMN_2308903 | -0,65 | 11,08 | -6,14 | 3,18E-06 | 1,49E-02 | 4,45 | ETFDH | 2110 |
| ILMN_1795336 | -0,66 | 9,03 | -3,97 | 6,27E-04 | 6,97E-02 | -0,25 | DGKH | 160851 |
| ILMN_2095597 | -0,66 | 8,49 | -4,10 | 4,59E-04 | 6,35E-02 | 0,03 | GUSBP11 | 91316 |
| ILMN_2398184 | -0,67 | 8,92 | -3,65 | 1,39E-03 | 9,43E-02 | -0,96 | HNF1B | 6928 |
| ILMN_1668411 | -0,67 | 8,44 | -4,04 | 5,32E-04 | 6,62E-02 | -0,10 | PPAP2B | 8613 |
| ILMN_1662154 | -0,67 | 8,93 | -3,75 | 1,07E-03 | 8,55E-02 | -0,73 | HRSP12 | 10247 |
| ILMN_1724021 | -0,67 | 8,81 | -3,62 | 1,49E-03 | 9,74E-02 | -1,03 | KIAA1211L | 343990 |
| ILMN_1722829 | -0,67 | 8,36 | -3,64 | 1,41E-03 | 9,53E-02 | -0,97 | RBM47 | 54502 |
| ILMN_1810172 | -0,67 | 8,08 | -3,96 | 6,39E-04 | 6,97E-02 | -0,26 | HRC | 3270 |
| ILMN_2374340 | -0,68 | 9,69 | -3,69 | 1,26E-03 | 8,99E-02 | -0,87 | PPAP2B | 8613 |
| ILMN_1771841 | -0,68 | 10,77 | -3,81 | 9,21E-04 | 8,02E-02 | -0,59 | ITGA8 | 8516 |
| ILMN_1682636 | -0,68 | 9,70 | -4,44 | 1,95E-04 | 4,61E-02 | 0,80 | D2HGDH | 728294 |
| ILMN_1759097 | -0,68 | 9,34 | -3,61 | 1,52E-03 | 9,79E-02 | -1,04 | PXMP2 | 5827 |
| ILMN_2075065 | -0,69 | 8,92 | -5,02 | 4,76E-05 | 3,16E-02 | 2,06 | CCDC183-AS1 | 1E+08 |
| ILMN_1734653 | -0,70 | 10,77 | -4,08 | 4,74E-04 | 6,37E-02 | 0,00 | NA | 92017 |
| ILMN_1701967 | -0,70 | 9,62 | -4,05 | 5,15E-04 | 6,53E-02 | -0,07 | SOX13 | 9580 |
| ILMN_1803030 | -0,70 | 8,44 | -3,90 | 7,43E-04 | 7,34E-02 | -0,40 | PKP4 | 8502 |
| ILMN_2120210 | -0,71 | 7,95 | -3,87 | 8,09E-04 | 7,54E-02 | -0,48 | CXCL12 | 6387 |
| ILMN_2082273 | -0,71 | 9,95 | -3,75 | 1,08E-03 | 8,55E-02 | -0,73 | LRRK2 | 120892 |
| ILMN_2399919 | -0,71 | 8,07 | -4,42 | 2,09E-04 | 4,74E-02 | 0,74 | KCNK5 | 8645 |
| ILMN_2306033 | -0,72 | 7,99 | -3,71 | 1,18E-03 | 8,76E-02 | -0,82 | IDNK | 414328 |
| ILMN_1764109 | -0,72 | 10,05 | -3,87 | 7,95E-04 | 7,44E-02 | -0,46 | CD34 | 947 |
| ILMN_1706505 | -0,72 | 7,68 | -3,72 | 1,16E-03 | 8,74E-02 | -0,80 | VIP | 7432 |
| ILMN_1809291 | -0,73 | 8,71 | -4,08 | 4,82E-04 | 6,42E-02 | -0,01 | TJP2 | 9414 |
| ILMN_2054607 | -0,73 | 7,16 | -5,12 | 3,71E-05 | 2,72E-02 | 2,28 | SLC6A13 | 6540 |
| ILMN_2216815 | -0,74 | 8,52 | -3,65 | 1,36E-03 | 9,34E-02 | -0,94 | CAPN3 | 825 |
| ILMN_1725172 | -0,74 | 8,80 | -4,51 | 1,67E-04 | 4,57E-02 | 0,94 | FBXW2 | 26190 |
| ILMN_1701308 | -0,75 | 7,66 | -3,96 | 6,37E-04 | 6,97E-02 | -0,26 | NEBL | 10529 |
| ILMN_1682737 | -0,75 | 7,90 | -3,94 | 6,74E-04 | 7,08E-02 | -0,31 | PANK1 | 53354 |
| ILMN_2232854 | -0,77 | 7,94 | -4,33 | 2,56E-04 | 4,94E-02 | 0,55 | PLLP | 51090 |
| ILMN_1701507 | -0,77 | 9,98 | -4,33 | 2,58E-04 | 4,95E-02 | 0,55 | ENOSF1 | 55556 |
| ILMN_1737096 | -0,77 | 8,57 | -4,89 | 6,56E-05 | 3,48E-02 | 1,77 | TPRN | 286262 |
| ILMN_1713529 | -0,77 | 7,73 | -4,83 | 7,54E-05 | 3,63E-02 | 1,65 | ALDH3A2 | 224 |
| ILMN_1712719 | -0,78 | 9,25 | -4,08 | 4,75E-04 | 6,37E-02 | 0,00 | SOX6 | 55553 |
| ILMN_1693192 | -0,78 | 10,59 | -3,85 | 8,41E-04 | 7,67E-02 | -0,51 | ABHD6 | 57406 |
| ILMN_1791511 | -0,79 | 9,82 | -4,34 | 2,53E-04 | 4,93E-02 | 0,57 | PCCA | 5095 |
| ILMN_3245611 | -0,79 | 8,19 | -3,82 | 9,17E-04 | 8,00E-02 | -0,59 | CATSPER3 | 347732 |
| ILMN_1684368 | -0,79 | 9,27 | -4,07 | 4,89E-04 | 6,46E-02 | -0,03 | ECI2 | 10455 |
| ILMN_1757129 | -0,80 | 9,42 | -4,52 | 1,64E-04 | 4,57E-02 | 0,96 | ACAT1 | 38 |
| ILMN_1801833 | -0,81 | 8,31 | -3,70 | 1,21E-03 | 8,78E-02 | -0,84 | ABAT | 18 |
| ILMN_1789653 | -0,81 | 10,06 | -5,00 | 5,04E-05 | 3,16E-02 | 2,01 | CLCN5 | 1184 |
| ILMN_1701562 | -0,82 | 10,67 | -4,10 | 4,52E-04 | 6,30E-02 | 0,05 | ACAA2 | 10449 |
| ILMN_2049184 | -0,82 | 7,75 | -5,15 | 3,47E-05 | 2,72E-02 | 2,34 | PDZK1 | 5174 |
| ILMN_1713901 | -0,83 | 9,41 | -3,70 | 1,23E-03 | 8,85E-02 | -0,85 | RASIP1 | 54922 |
| ILMN_1800091 | -0,84 | 7,71 | -3,90 | 7,55E-04 | 7,34E-02 | -0,42 | TMEM125 | 128218 |
| ILMN_2171384 | -0,84 | 10,56 | -3,97 | 6,24E-04 | 6,97E-02 | -0,24 | TNFRSF21 | 27242 |
| ILMN_1718852 | -0,85 | 7,27 | -3,72 | 1,16E-03 | 8,74E-02 | -0,80 | AGPAT2 | 10555 |
| ILMN_1705750 | -0,86 | 10,57 | -3,63 | 1,44E-03 | 9,61E-02 | -0,99 | AUTS2 | 26053 |
| ILMN_1693230 | -0,86 | 8,03 | -4,56 | 1,48E-04 | 4,49E-02 | 1,05 | PLS1 | 5357 |
| ILMN_1662147 | -0,87 | 8,62 | -3,83 | 8,91E-04 | 7,87E-02 | -0,56 | CHDH | 55349 |
| ILMN_1720373 | -0,87 | 10,30 | -3,98 | 6,15E-04 | 6,97E-02 | -0,23 | RENBP | 5973 |
| ILMN_2228180 | -0,90 | 9,61 | -3,64 | 1,41E-03 | 9,53E-02 | -0,98 | PTPRB | 5787 |
| ILMN_1796628 | -0,90 | 11,07 | -4,53 | 1,58E-04 | 4,57E-02 | 0,99 | GOT1 | 2805 |
| ILMN_1784948 | -0,91 | 8,37 | -4,06 | 5,02E-04 | 6,49E-02 | -0,05 | ANK3 | 288 |
| ILMN_1700831 | -0,92 | 9,25 | -4,08 | 4,74E-04 | 6,37E-02 | 0,00 | MYOZ1 | 58529 |
| ILMN_2289593 | -0,92 | 7,75 | -4,63 | 1,24E-04 | 4,29E-02 | 1,20 | PAX2 | 5076 |
| ILMN_3246401 | -0,92 | 9,14 | -4,39 | 2,24E-04 | 4,81E-02 | 0,68 | NOXA1 | 10811 |
| ILMN_1653871 | -0,93 | 10,22 | -3,74 | 1,10E-03 | 8,56E-02 | -0,75 | EPHX2 | 2053 |
| ILMN_1678842 | -0,94 | 10,53 | -5,38 | 1,99E-05 | 2,22E-02 | 2,84 | AUH | 549 |
| ILMN_1791328 | -0,94 | 10,45 | -4,05 | 5,12E-04 | 6,52E-02 | -0,07 | EFHD1 | 80303 |
| ILMN_1672589 | -0,94 | 9,62 | -3,93 | 6,87E-04 | 7,12E-02 | -0,33 | PTH1R | 5745 |
| ILMN_1806037 | -0,94 | 11,49 | -3,62 | 1,49E-03 | 9,74E-02 | -1,02 | UGT2B7 | 7364 |
| ILMN_1767324 | -0,94 | 8,41 | -5,18 | 3,22E-05 | 2,72E-02 | 2,41 | ANK3 | 288 |
| ILMN_1677603 | -0,94 | 9,25 | -3,99 | 6,02E-04 | 6,89E-02 | -0,21 | LMO7 | 4008 |
| ILMN_1770479 | -0,96 | 12,22 | -4,48 | 1,79E-04 | 4,61E-02 | 0,88 | AIF1L | 83543 |
| ILMN_1661875 | -0,96 | 11,97 | -4,29 | 2,85E-04 | 5,10E-02 | 0,46 | FXYD2 | 486 |
| ILMN_1755897 | -0,97 | 7,38 | -4,71 | 1,02E-04 | 3,93E-02 | 1,38 | SLC27A2 | 11001 |
| ILMN_1795166 | -0,98 | 9,04 | -3,75 | 1,09E-03 | 8,55E-02 | -0,74 | MSRA | 4482 |
| ILMN_1779448 | -0,98 | 7,82 | -5,24 | 2,76E-05 | 2,69E-02 | 2,55 | HYAL1 | 3373 |
| ILMN_2231911 | -0,99 | 8,77 | -4,12 | 4,31E-04 | 6,17E-02 | 0,09 | PLCL1 | 5334 |
| ILMN_1654571 | -0,99 | 8,10 | -3,74 | 1,12E-03 | 8,60E-02 | -0,76 | DNASE1L3 | 1776 |
| ILMN_1709237 | -1,00 | 7,80 | -4,27 | 3,00E-04 | 5,22E-02 | 0,41 | PKHD1 | 5314 |
| ILMN_1659905 | -1,00 | 10,10 | -3,66 | 1,35E-03 | 9,29E-02 | -0,94 | PBLD | 64081 |
| ILMN_1667361 | -1,00 | 10,54 | -3,92 | 7,05E-04 | 7,22E-02 | -0,35 | ARHGAP24 | 83478 |
| ILMN_1686664 | -1,01 | 9,80 | -3,60 | 1,56E-03 | 9,95E-02 | -1,06 | TMEM88 | 92162 |
| ILMN_1799589 | -1,02 | 9,22 | -3,63 | 1,44E-03 | 9,61E-02 | -0,99 | TMEM176A | 55365 |
| ILMN_2355831 | -1,02 | 8,18 | -4,05 | 5,21E-04 | 6,59E-02 | -0,08 | MAP7 | 9053 |
| ILMN_1734365 | -1,03 | 10,34 | -3,94 | 6,83E-04 | 7,12E-02 | -0,32 | SEMA6A | 57556 |
| ILMN_1812570 | -1,03 | 9,08 | -4,01 | 5,66E-04 | 6,73E-02 | -0,16 | CLDN10 | 9071 |
| ILMN_1731157 | -1,04 | 9,91 | -4,16 | 3,97E-04 | 5,98E-02 | 0,16 | EHHADH | 1962 |
| ILMN_1731353 | -1,05 | 7,91 | -4,48 | 1,78E-04 | 4,61E-02 | 0,88 | PKLR | 5313 |
| ILMN_2188451 | -1,05 | 8,58 | -3,94 | 6,82E-04 | 7,12E-02 | -0,32 | MAP7 | 9053 |
| ILMN_2390609 | -1,06 | 10,43 | -4,96 | 5,46E-05 | 3,28E-02 | 1,94 | CYP4V2 | 285440 |
| ILMN_1787897 | -1,06 | 9,04 | -4,09 | 4,64E-04 | 6,37E-02 | 0,02 | TSPAN7 | 7102 |
| ILMN_1753111 | -1,08 | 10,78 | -3,61 | 1,52E-03 | 9,79E-02 | -1,04 | MLXIPL | 51085 |
| ILMN_1656145 | -1,08 | 10,38 | -3,61 | 1,51E-03 | 9,78E-02 | -1,03 | RGS5 | 8490 |
| ILMN_1672102 | -1,08 | 10,10 | -3,96 | 6,42E-04 | 6,97E-02 | -0,27 | RCAN2 | 10231 |
| ILMN_1731446 | -1,08 | 7,91 | -3,93 | 6,88E-04 | 7,12E-02 | -0,33 | NOS1 | 4842 |
| ILMN_1708151 | -1,12 | 9,48 | -4,15 | 4,08E-04 | 6,07E-02 | 0,14 | HLF | 3131 |
| ILMN_2169261 | -1,12 | 7,34 | -4,36 | 2,39E-04 | 4,81E-02 | 0,62 | SLC6A19 | 340024 |
| ILMN_1661076 | -1,12 | 7,48 | -4,81 | 7,86E-05 | 3,68E-02 | 1,61 | SLC22A12 | 116085 |
| ILMN_1676289 | -1,12 | 11,45 | -4,21 | 3,52E-04 | 5,64E-02 | 0,27 | CDH16 | 1014 |
| ILMN_1775170 | -1,13 | 9,77 | -3,70 | 1,21E-03 | 8,78E-02 | -0,84 | PTER | 9317 |
| ILMN_1785393 | -1,14 | 8,78 | -4,94 | 5,84E-05 | 3,28E-02 | 1,88 | MAPK15 | 225689 |
| ILMN_1706643 | -1,14 | 8,38 | -3,86 | 8,33E-04 | 7,64E-02 | -0,50 | SLC16A12 | 387700 |
| ILMN_1780057 | -1,14 | 9,27 | -4,11 | 4,45E-04 | 6,23E-02 | 0,06 | RAB3IP | 117177 |
| ILMN_2135321 | -1,15 | 7,51 | -3,62 | 1,49E-03 | 9,74E-02 | -1,03 | PCK1 | 5105 |
| ILMN_1694426 | -1,20 | 8,64 | -3,75 | 1,08E-03 | 8,55E-02 | -0,74 | GLYAT | 10249 |
| ILMN_2074044 | -1,24 | 7,52 | -4,36 | 2,42E-04 | 4,83E-02 | 0,60 | SLC22A12 | 116085 |
| ILMN_1773389 | -1,25 | 10,30 | -3,76 | 1,04E-03 | 8,47E-02 | -0,70 | GATM | 2628 |
| ILMN_1749081 | -1,26 | 8,76 | -3,90 | 7,51E-04 | 7,34E-02 | -0,41 | TMEM171 | 134285 |
| ILMN_2377430 | -1,26 | 9,62 | -3,91 | 7,27E-04 | 7,32E-02 | -0,38 | SLC7A9 | 11136 |
| ILMN_1699695 | -1,26 | 8,49 | -4,67 | 1,11E-04 | 3,96E-02 | 1,31 | UGT2B17 | 7367 |
| ILMN_1687301 | -1,27 | 8,90 | -3,63 | 1,44E-03 | 9,61E-02 | -0,99 | CIT | 11113 |
| ILMN_1685709 | -1,30 | 8,61 | -4,26 | 3,08E-04 | 5,32E-02 | 0,39 | CYS1 | 192668 |
| ILMN_3251383 | -1,34 | 8,37 | -4,80 | 8,05E-05 | 3,68E-02 | 1,59 | FUT6 | 2528 |
| ILMN_1755657 | -1,35 | 8,44 | -4,19 | 3,63E-04 | 5,71E-02 | 0,24 | C2orf40 | 84417 |
| ILMN_2408543 | -1,36 | 9,46 | -3,68 | 1,29E-03 | 9,09E-02 | -0,89 | SLC28A1 | 9154 |
| ILMN_1749032 | -1,40 | 11,37 | -4,48 | 1,78E-04 | 4,61E-02 | 0,88 | ESPN | 83715 |
| ILMN_1797236 | -1,44 | 8,43 | -4,36 | 2,38E-04 | 4,81E-02 | 0,62 | AIF1L | 83543 |
| ILMN_1694535 | -1,45 | 11,36 | -5,43 | 1,75E-05 | 2,20E-02 | 2,95 | VIPR1 | 7433 |
| ILMN_1660199 | -1,46 | 11,54 | -4,08 | 4,74E-04 | 6,37E-02 | 0,00 | LRP2 | 4036 |
| ILMN_1690327 | -1,46 | 9,06 | -4,28 | 2,96E-04 | 5,21E-02 | 0,42 | PLCH2 | 9651 |
| ILMN_1786065 | -1,46 | 8,91 | -4,58 | 1,41E-04 | 4,49E-02 | 1,09 | FREM2 | 341640 |
| ILMN_1805104 | -1,46 | 7,81 | -5,15 | 3,47E-05 | 2,72E-02 | 2,34 | LRRC19 | 64922 |
| ILMN_1800008 | -1,47 | 9,08 | -3,96 | 6,39E-04 | 6,97E-02 | -0,26 | PHYHIPL | 84457 |
| ILMN_1815023 | -1,48 | 8,88 | -4,64 | 1,20E-04 | 4,23E-02 | 1,23 | EMX2 | 2018 |
| ILMN_1799139 | -1,48 | 8,87 | -4,34 | 2,53E-04 | 4,93E-02 | 0,57 | PKHD1 | 5314 |
| ILMN_1714170 | -1,49 | 8,64 | -4,10 | 4,62E-04 | 6,37E-02 | 0,03 | PKHD1 | 5314 |
| ILMN_1761131 | -1,50 | 10,86 | -5,00 | 5,02E-05 | 3,16E-02 | 2,01 | MYL3 | 4634 |
| ILMN_1686313 | -1,52 | 9,41 | -4,39 | 2,25E-04 | 4,81E-02 | 0,67 | FBP1 | 2203 |
| ILMN_1693961 | -1,52 | 10,01 | -5,51 | 1,45E-05 | 2,15E-02 | 3,12 | AQP1 | 358 |
| ILMN_1714384 | -1,52 | 9,82 | -3,88 | 7,79E-04 | 7,38E-02 | -0,44 | BBOX1 | 8424 |
| ILMN_3244286 | -1,58 | 9,55 | -4,02 | 5,50E-04 | 6,67E-02 | -0,13 | SLC39A5 | 283375 |
| ILMN_1784459 | -1,59 | 9,04 | -3,65 | 1,39E-03 | 9,43E-02 | -0,96 | CGN | 57530 |
| ILMN_1706344 | -1,60 | 9,09 | -3,73 | 1,13E-03 | 8,66E-02 | -0,78 | SLC17A3 | 10786 |
| ILMN_1663327 | -1,60 | 8,19 | -5,64 | 1,05E-05 | 2,09E-02 | 3,40 | CDHR2 | 54825 |
| ILMN_2401641 | -1,63 | 8,62 | -3,68 | 1,29E-03 | 9,09E-02 | -0,89 | DHDH | 27294 |
| ILMN_1726266 | -1,64 | 8,94 | -5,00 | 4,98E-05 | 3,16E-02 | 2,02 | TINAG | 27283 |
| ILMN_1664283 | -1,65 | 9,48 | -4,40 | 2,18E-04 | 4,79E-02 | 0,70 | OGDHL | 55753 |
| ILMN_1803676 | -1,66 | 8,09 | -5,67 | 9,94E-06 | 2,09E-02 | 3,45 | ACE2 | 59272 |
| ILMN_2082865 | -1,67 | 8,37 | -5,89 | 5,77E-06 | 1,70E-02 | 3,93 | SLC5A10 | 125206 |
| ILMN_1783946 | -1,68 | 7,82 | -3,65 | 1,38E-03 | 9,41E-02 | -0,96 | SLC13A2 | 9058 |
| ILMN_1783497 | -1,72 | 9,90 | -3,68 | 1,27E-03 | 9,04E-02 | -0,88 | PPARGC1A | 10891 |
| ILMN_1675927 | -1,75 | 10,53 | -4,72 | 9,92E-05 | 3,93E-02 | 1,41 | PAX2 | 5076 |
| ILMN_2337492 | -1,75 | 8,70 | -3,83 | 8,80E-04 | 7,82E-02 | -0,55 | FXYD2 | 486 |
| ILMN_3251567 | -1,75 | 9,09 | -4,80 | 8,15E-05 | 3,68E-02 | 1,58 | FLRT3 | 23767 |
| ILMN_1687971 | -1,80 | 9,07 | -4,80 | 8,16E-05 | 3,68E-02 | 1,58 | NPR3 | 4883 |
| ILMN_1683492 | -1,81 | 9,37 | -4,74 | 9,38E-05 | 3,76E-02 | 1,45 | GBA3 | 57733 |
| ILMN_1707710 | -1,81 | 9,68 | -3,95 | 6,66E-04 | 7,04E-02 | -0,30 | ACSM2B | 348158 |
| ILMN_2384561 | -1,83 | 10,69 | -3,78 | 1,00E-03 | 8,22E-02 | -0,67 | CA4 | 762 |
| ILMN_1732538 | -1,90 | 10,12 | -4,20 | 3,54E-04 | 5,64E-02 | 0,27 | ACSM2B | 348158 |
| ILMN_1707807 | -1,91 | 8,62 | -6,26 | 2,43E-06 | 1,40E-02 | 4,69 | SLC5A10 | 125206 |
| ILMN_1794638 | -1,97 | 9,97 | -3,71 | 1,20E-03 | 8,77E-02 | -0,83 | FTCD | 10841 |
| ILMN_2341229 | -2,00 | 8,85 | -5,64 | 1,05E-05 | 2,09E-02 | 3,41 | HAO2 | 51179 |
| ILMN_1791306 | -2,00 | 9,73 | -3,62 | 1,47E-03 | 9,73E-02 | -1,01 | PKLR | 5313 |
| ILMN_1766918 | -2,02 | 11,08 | -4,19 | 3,69E-04 | 5,71E-02 | 0,23 | NAT8 | 9027 |
| ILMN_2226015 | -2,06 | 10,17 | -4,23 | 3,34E-04 | 5,49E-02 | 0,32 | FTCD | 10841 |
| ILMN_2406313 | -2,09 | 9,46 | -4,83 | 7,59E-05 | 3,63E-02 | 1,64 | SLC22A2 | 6582 |
| ILMN_2311497 | -2,11 | 10,01 | -5,56 | 1,28E-05 | 2,09E-02 | 3,23 | TMEM27 | 57393 |
| ILMN_1791447 | -2,15 | 9,90 | -4,44 | 1,97E-04 | 4,62E-02 | 0,79 | GBA3 | 57733 |
| ILMN_1661325 | -2,20 | 9,90 | -5,62 | 1,11E-05 | 2,09E-02 | 3,35 | TRPM3 | 80036 |
| ILMN_1683755 | -2,22 | 8,79 | -4,39 | 2,23E-04 | 4,81E-02 | 0,68 | LOC389332 | 389332 |
| ILMN_1670539 | -2,27 | 10,82 | -4,58 | 1,41E-04 | 4,49E-02 | 1,09 | DDC | 1644 |
| ILMN_3244176 | -2,32 | 10,11 | -4,76 | 9,08E-05 | 3,76E-02 | 1,48 | SLC22A2 | 6582 |
| ILMN_3244698 | -2,36 | 8,42 | -4,37 | 2,35E-04 | 4,81E-02 | 0,63 | ANGPTL3 | 27329 |
| ILMN_2289433 | -2,45 | 11,43 | -5,03 | 4,67E-05 | 3,16E-02 | 2,08 | AOC1 | 26 |
| ILMN_1750250 | -2,51 | 9,57 | -5,38 | 1,97E-05 | 2,22E-02 | 2,84 | HAO2 | 51179 |
| ILMN_1799015 | -2,88 | 10,07 | -6,88 | 5,83E-07 | 5,03E-03 | 5,92 | MIOX | 55586 |
| ILMN_2206272 | -3,00 | 9,22 | -4,46 | 1,87E-04 | 4,61E-02 | 0,84 | SLC22A6 | 9356 |
| ILMN_1660306 | -3,18 | 9,10 | -7,63 | 1,12E-07 | 2,99E-03 | 7,33 | SLC22A12 | 116085 |
| ILMN_2388800 | -3,40 | 9,14 | -5,59 | 1,20E-05 | 2,09E-02 | 3,29 | ALDOB | 229 |
| ILMN_2141482 | 0,09 | 6,65 | 4,14 | 4,17E-04 | 6,12E-02 | 0,12 | NA | 391004 |
| ILMN_1738773 | 0,10 | 6,84 | 3,74 | 1,09E-03 | 8,55E-02 | -0,75 | SNORD42B | 22897 |
| ILMN_3306730 | 0,11 | 6,72 | 3,72 | 1,16E-03 | 8,74E-02 | -0,80 | ACTR3C | 653857 |
| ILMN_1776121 | 0,12 | 6,70 | 4,41 | 2,15E-04 | 4,79E-02 | 0,71 | PCSK1 | 5122 |
| ILMN_1807633 | 0,12 | 6,82 | 3,89 | 7,65E-04 | 7,34E-02 | -0,43 | LRRC15 | 131578 |
| ILMN_1684726 | 0,13 | 6,65 | 3,83 | 8,80E-04 | 7,82E-02 | -0,55 | CHRNB2 | 1141 |
| ILMN_1723978 | 0,14 | 6,73 | 3,71 | 1,18E-03 | 8,74E-02 | -0,81 | PDCL2 | 132954 |
| ILMN_1681583 | 0,15 | 6,85 | 3,80 | 9,50E-04 | 8,11E-02 | -0,62 | NA | 84641 |
| ILMN_2408683 | 0,15 | 6,70 | 4,31 | 2,73E-04 | 5,01E-02 | 0,50 | PRR5 | 55615 |
| ILMN_3235709 | 0,17 | 6,85 | 4,49 | 1,74E-04 | 4,61E-02 | 0,90 | TCP1 | 6950 |
| ILMN_1721022 | 0,18 | 7,01 | 4,09 | 4,67E-04 | 6,37E-02 | 0,02 | GTDC1 | 79712 |
| ILMN_3240541 | 0,19 | 7,16 | 3,72 | 1,16E-03 | 8,74E-02 | -0,80 | SKIL | 6498 |
| ILMN_1696029 | 0,20 | 6,67 | 4,51 | 1,67E-04 | 4,57E-02 | 0,94 | HCAR3 | 8843 |
| ILMN_3235718 | 0,20 | 6,80 | 4,57 | 1,44E-04 | 4,49E-02 | 1,07 | LPAL2 | 80350 |
| ILMN_1756650 | 0,21 | 6,71 | 4,22 | 3,44E-04 | 5,59E-02 | 0,29 | VSX1 | 30813 |
| ILMN_1758034 | 0,22 | 6,80 | 4,03 | 5,47E-04 | 6,67E-02 | -0,13 | ICAM5 | 7087 |
| ILMN_1735151 | 0,23 | 7,33 | 3,70 | 1,21E-03 | 8,77E-02 | -0,83 | NA | 649 |
| ILMN_1751028 | 0,24 | 6,95 | 4,31 | 2,72E-04 | 5,01E-02 | 0,50 | SEMA4B | 10509 |
| ILMN_1710514 | 0,24 | 6,93 | 4,22 | 3,43E-04 | 5,59E-02 | 0,29 | ICAM1 | 3383 |
| ILMN_1797557 | 0,24 | 7,06 | 4,10 | 4,57E-04 | 6,35E-02 | 0,04 | SHISA4 | 149345 |
| ILMN_2293374 | 0,25 | 6,91 | 3,89 | 7,66E-04 | 7,34E-02 | -0,43 | CHEK1 | 1111 |
| ILMN_3308848 | 0,25 | 6,71 | 3,73 | 1,14E-03 | 8,69E-02 | -0,79 | NPC1L1 | 29881 |
| ILMN_1786139 | 0,28 | 6,76 | 4,93 | 5,90E-05 | 3,28E-02 | 1,87 | HCAR2 | 338442 |
| ILMN_1785933 | 0,28 | 8,72 | 3,72 | 1,17E-03 | 8,74E-02 | -0,81 | ZFYVE1 | 53349 |
| ILMN_1785427 | 0,28 | 7,13 | 3,91 | 7,35E-04 | 7,32E-02 | -0,39 | TBX6 | 6911 |
| ILMN_1766221 | 0,29 | 6,80 | 3,71 | 1,17E-03 | 8,74E-02 | -0,81 | FOXA1 | 3169 |
| ILMN_1778941 | 0,29 | 6,86 | 4,47 | 1,86E-04 | 4,61E-02 | 0,84 | ABCA12 | 26154 |
| ILMN_1745116 | 0,30 | 7,10 | 3,92 | 7,14E-04 | 7,26E-02 | -0,36 | RASSF2 | 9770 |
| ILMN_1739161 | 0,31 | 6,78 | 3,76 | 1,05E-03 | 8,50E-02 | -0,71 | RTBDN | 83546 |
| ILMN_1752520 | 0,31 | 7,17 | 3,81 | 9,35E-04 | 8,07E-02 | -0,61 | FANCI | 55215 |
| ILMN_1684873 | 0,31 | 6,87 | 4,04 | 5,33E-04 | 6,62E-02 | -0,10 | COL10A1 | 1300 |
| ILMN_1717853 | 0,32 | 6,90 | 4,50 | 1,71E-04 | 4,59E-02 | 0,92 | FN1 | 2335 |
| ILMN_1656560 | 0,32 | 6,86 | 3,65 | 1,37E-03 | 9,37E-02 | -0,95 | ADAMTS14 | 140766 |
| ILMN_1811345 | 0,32 | 6,94 | 4,53 | 1,57E-04 | 4,57E-02 | 0,99 | DGKG | 1608 |
| ILMN_1697820 | 0,32 | 7,72 | 3,73 | 1,12E-03 | 8,61E-02 | -0,77 | UBTF | 7343 |
| ILMN_1655595 | 0,32 | 8,02 | 3,67 | 1,31E-03 | 9,14E-02 | -0,91 | ARF4 | 378 |
| ILMN_1676545 | 0,32 | 11,42 | 3,89 | 7,72E-04 | 7,35E-02 | -0,43 | FNDC3B | 64778 |
| ILMN_1814327 | 0,33 | 7,41 | 3,68 | 1,26E-03 | 9,01E-02 | -0,88 | SEC24D | 9871 |
| ILMN_1666967 | 0,33 | 7,60 | 4,71 | 1,03E-04 | 3,93E-02 | 1,37 | SMARCD2 | 6603 |
| ILMN_1675756 | 0,33 | 7,03 | 3,66 | 1,35E-03 | 9,28E-02 | -0,93 | SLC35A2 | 7355 |
| ILMN_2256687 | 0,33 | 7,44 | 4,48 | 1,81E-04 | 4,61E-02 | 0,87 | BTG3 | 10950 |
| ILMN_1737025 | 0,33 | 7,92 | 3,90 | 7,45E-04 | 7,34E-02 | -0,40 | PLIN3 | 10226 |
| ILMN_3247139 | 0,34 | 7,09 | 5,15 | 3,44E-05 | 2,72E-02 | 2,35 | LENG9 | 94059 |
| ILMN_1708743 | 0,34 | 7,03 | 3,67 | 1,31E-03 | 9,13E-02 | -0,90 | LUM | 4060 |
| ILMN_1667298 | 0,34 | 8,49 | 3,79 | 9,71E-04 | 8,19E-02 | -0,64 | INTS1 | 26173 |
| ILMN_1694070 | 0,35 | 9,39 | 3,60 | 1,56E-03 | 9,96E-02 | -1,07 | PPP1R14B | 26472 |
| ILMN_1727194 | 0,36 | 7,38 | 4,61 | 1,31E-04 | 4,46E-02 | 1,16 | TRAM2 | 9697 |
| ILMN_1775708 | 0,36 | 11,41 | 3,90 | 7,48E-04 | 7,34E-02 | -0,41 | CARS | 833 |
| ILMN_1762810 | 0,37 | 8,18 | 3,67 | 1,31E-03 | 9,14E-02 | -0,91 | GMPPA | 29926 |
| ILMN_1750912 | 0,37 | 7,70 | 3,88 | 7,81E-04 | 7,38E-02 | -0,45 | C11orf24 | 53838 |
| ILMN_1685917 | 0,38 | 8,97 | 4,52 | 1,60E-04 | 4,57E-02 | 0,98 | GNL3 | 26354 |
| ILMN_1663347 | 0,39 | 7,02 | 3,66 | 1,34E-03 | 9,24E-02 | -0,93 | MANEAL | 149175 |
| ILMN_2201678 | 0,40 | 8,50 | 4,00 | 5,90E-04 | 6,83E-02 | -0,19 | EXT2 | 2132 |
| ILMN_2169152 | 0,40 | 6,82 | 3,78 | 9,95E-04 | 8,21E-02 | -0,66 | ATN1 | 1822 |
| ILMN_1755281 | 0,40 | 12,33 | 3,62 | 1,49E-03 | 9,74E-02 | -1,02 | NME4 | 4833 |
| ILMN_1739667 | 0,41 | 10,19 | 4,19 | 3,68E-04 | 5,71E-02 | 0,23 | TPD52L2 | 7165 |
| ILMN_1794825 | 0,41 | 6,88 | 3,83 | 8,76E-04 | 7,82E-02 | -0,55 | COL11A1 | 1301 |
| ILMN_1696432 | 0,41 | 10,29 | 4,43 | 2,00E-04 | 4,62E-02 | 0,77 | ST5 | 6764 |
| ILMN_1676515 | 0,42 | 6,96 | 3,91 | 7,21E-04 | 7,31E-02 | -0,37 | LILRA5 | 353514 |
| ILMN_1668507 | 0,42 | 7,53 | 5,11 | 3,78E-05 | 2,72E-02 | 2,26 | CARS | 833 |
| ILMN_1808537 | 0,43 | 8,21 | 3,61 | 1,50E-03 | 9,74E-02 | -1,03 | NOP2 | 4839 |
| ILMN_1802093 | 0,43 | 7,22 | 3,83 | 8,97E-04 | 7,88E-02 | -0,57 | UXT | 8409 |
| ILMN_2163873 | 0,44 | 7,20 | 4,83 | 7,47E-05 | 3,63E-02 | 1,66 | SH3PXD2B | 285590 |
| ILMN_1719547 | 0,44 | 7,14 | 3,78 | 9,97E-04 | 8,21E-02 | -0,66 | PRR19 | 284338 |
| ILMN_1669465 | 0,45 | 9,26 | 3,80 | 9,56E-04 | 8,14E-02 | -0,63 | CCDC28B | 79140 |
| ILMN_1683059 | 0,46 | 8,50 | 3,82 | 9,13E-04 | 7,99E-02 | -0,58 | EDEM2 | 55741 |
| ILMN_1663538 | 0,46 | 8,86 | 4,32 | 2,65E-04 | 5,01E-02 | 0,53 | ELK1 | 2002 |
| ILMN_1738767 | 0,46 | 7,22 | 4,19 | 3,64E-04 | 5,71E-02 | 0,24 | KCNT2 | 343450 |
| ILMN_1687306 | 0,47 | 7,27 | 3,96 | 6,46E-04 | 6,97E-02 | -0,28 | MYOZ3 | 91977 |
| ILMN_1778337 | 0,47 | 10,42 | 3,91 | 7,32E-04 | 7,32E-02 | -0,39 | TFG | 10342 |
| ILMN_1664369 | 0,48 | 7,36 | 4,45 | 1,93E-04 | 4,61E-02 | 0,81 | LOC155060 | 155060 |
| ILMN_3310080 | 0,49 | 9,21 | 4,87 | 6,81E-05 | 3,56E-02 | 1,74 | QSOX1 | 5768 |
| ILMN_2343048 | 0,49 | 6,89 | 3,91 | 7,30E-04 | 7,32E-02 | -0,38 | EYA1 | 2138 |
| ILMN_1741133 | 0,49 | 7,19 | 4,19 | 3,69E-04 | 5,71E-02 | 0,23 | RPL39L | 116832 |
| ILMN_1663407 | 0,49 | 7,05 | 4,00 | 5,82E-04 | 6,78E-02 | -0,18 | RARRES1 | 5918 |
| ILMN_1683112 | 0,50 | 9,51 | 3,75 | 1,08E-03 | 8,55E-02 | -0,73 | SNORD12B | 1E+08 |
| ILMN_1711030 | 0,50 | 7,41 | 4,30 | 2,80E-04 | 5,08E-02 | 0,47 | PTGFRN | 5738 |
| ILMN_1704702 | 0,50 | 7,14 | 3,78 | 9,94E-04 | 8,21E-02 | -0,66 | NA | 10406 |
| ILMN_1777665 | 0,50 | 7,47 | 3,95 | 6,66E-04 | 7,04E-02 | -0,30 | RTN2 | 6253 |
| ILMN_1687216 | 0,50 | 8,50 | 4,23 | 3,28E-04 | 5,43E-02 | 0,33 | EFTUD2 | 9343 |
| ILMN_1710092 | 0,50 | 9,83 | 3,62 | 1,47E-03 | 9,73E-02 | -1,01 | ARPC1A | 10552 |
| ILMN_2378100 | 0,51 | 7,88 | 3,90 | 7,46E-04 | 7,34E-02 | -0,40 | ABCB9 | 23457 |
| ILMN_1726466 | 0,51 | 11,24 | 3,88 | 7,77E-04 | 7,38E-02 | -0,44 | ARF4 | 378 |
| ILMN_1739576 | 0,51 | 8,57 | 3,79 | 9,84E-04 | 8,20E-02 | -0,65 | NA | 727866 |
| ILMN_1717877 | 0,51 | 9,46 | 3,78 | 1,01E-03 | 8,24E-02 | -0,67 | SNORD12B | 1E+08 |
| ILMN_2213297 | 0,52 | 7,59 | 4,59 | 1,36E-04 | 4,47E-02 | 1,12 | KCNT2 | 343450 |
| ILMN_1727043 | 0,53 | 10,26 | 4,06 | 5,01E-04 | 6,49E-02 | -0,05 | PLEC | 5339 |
| ILMN_1706031 | 0,53 | 7,34 | 4,06 | 4,98E-04 | 6,48E-02 | -0,04 | FSCN2 | 25794 |
| ILMN_1669113 | 0,54 | 8,03 | 3,73 | 1,13E-03 | 8,67E-02 | -0,78 | H2AFX | 3014 |
| ILMN_1696316 | 0,54 | 9,49 | 3,69 | 1,25E-03 | 8,95E-02 | -0,87 | P3H1 | 64175 |
| ILMN_1670130 | 0,54 | 9,67 | 4,29 | 2,85E-04 | 5,10E-02 | 0,46 | SRM | 6723 |
| ILMN_1693762 | 0,54 | 8,33 | 4,02 | 5,57E-04 | 6,71E-02 | -0,14 | ARID3A | 1820 |
| ILMN_1661337 | 0,54 | 9,32 | 3,90 | 7,51E-04 | 7,34E-02 | -0,41 | ATF5 | 22809 |
| ILMN_1664294 | 0,55 | 9,35 | 3,74 | 1,10E-03 | 8,56E-02 | -0,75 | COLGALT1 | 79709 |
| ILMN_1797367 | 0,55 | 7,23 | 4,12 | 4,34E-04 | 6,18E-02 | 0,08 | CYB5R2 | 51700 |
| ILMN_1785756 | 0,55 | 7,08 | 3,60 | 1,57E-03 | 9,96E-02 | -1,07 | PCBP3 | 54039 |
| ILMN_1801476 | 0,56 | 7,55 | 3,84 | 8,56E-04 | 7,73E-02 | -0,53 | MCM7 | 4176 |
| ILMN_1668608 | 0,56 | 10,00 | 3,67 | 1,30E-03 | 9,09E-02 | -0,90 | NME1 | 4830 |
| ILMN_1713247 | 0,56 | 8,28 | 4,25 | 3,12E-04 | 5,36E-02 | 0,38 | ABCB9 | 23457 |
| ILMN_1776857 | 0,58 | 7,59 | 3,83 | 8,77E-04 | 7,82E-02 | -0,55 | PLP2 | 5355 |
| ILMN_1685313 | 0,58 | 7,61 | 3,85 | 8,35E-04 | 7,64E-02 | -0,50 | INHBA | 3624 |
| ILMN_1749641 | 0,59 | 7,24 | 4,03 | 5,45E-04 | 6,66E-02 | -0,12 | FNDC1 | 84624 |
| ILMN_2053345 | 0,59 | 9,02 | 4,78 | 8,47E-05 | 3,74E-02 | 1,55 | IMPDH1 | 3614 |
| ILMN_3236704 | 0,59 | 7,62 | 3,67 | 1,32E-03 | 9,20E-02 | -0,92 | IDH1 | 3417 |
| ILMN_1658160 | 0,59 | 8,04 | 3,70 | 1,23E-03 | 8,84E-02 | -0,85 | JAK3 | 3718 |
| ILMN_1787378 | 0,60 | 8,35 | 3,90 | 7,44E-04 | 7,34E-02 | -0,40 | SRGN | 5552 |
| ILMN_1723884 | 0,60 | 8,51 | 4,24 | 3,26E-04 | 5,42E-02 | 0,34 | FSTL1 | 11167 |
| ILMN_1786024 | 0,60 | 7,50 | 3,78 | 1,01E-03 | 8,24E-02 | -0,67 | OTOF | 9381 |
| ILMN_1775328 | 0,60 | 7,33 | 4,67 | 1,11E-04 | 3,96E-02 | 1,30 | STXBP6 | 29091 |
| ILMN_2343047 | 0,60 | 7,24 | 4,05 | 5,12E-04 | 6,52E-02 | -0,07 | IL1RL2 | 8808 |
| ILMN_1759915 | 0,60 | 12,21 | 4,75 | 9,13E-05 | 3,76E-02 | 1,48 | SLC2A3 | 6515 |
| ILMN_1738819 | 0,60 | 10,56 | 4,92 | 6,01E-05 | 3,29E-02 | 1,85 | CALU | 813 |
| ILMN_1671568 | 0,60 | 7,72 | 5,31 | 2,36E-05 | 2,49E-02 | 2,68 | FAM114A1 | 92689 |
| ILMN_2373377 | 0,61 | 9,12 | 3,93 | 6,90E-04 | 7,12E-02 | -0,33 | NT5DC2 | 64943 |
| ILMN_1668714 | 0,61 | 7,64 | 3,97 | 6,25E-04 | 6,97E-02 | -0,25 | C17orf96 | 1E+08 |
| ILMN_1799120 | 0,62 | 12,98 | 4,02 | 5,57E-04 | 6,71E-02 | -0,14 | SERPINE2 | 5270 |
| ILMN_2309926 | 0,63 | 8,90 | 4,07 | 4,92E-04 | 6,46E-02 | -0,03 | SLFN11 | 91607 |
| ILMN_2077905 | 0,63 | 9,14 | 4,24 | 3,21E-04 | 5,42E-02 | 0,35 | ABHD12 | 26090 |
| ILMN_3244669 | 0,63 | 8,76 | 4,14 | 4,14E-04 | 6,10E-02 | 0,12 | B4GALT1 | 2683 |
| ILMN_1811468 | 0,64 | 8,35 | 4,70 | 1,05E-04 | 3,93E-02 | 1,36 | VKORC1 | 79001 |
| ILMN_1677851 | 0,64 | 8,37 | 4,29 | 2,89E-04 | 5,11E-02 | 0,45 | TOP1MT | 116447 |
| ILMN_1712413 | 0,64 | 9,48 | 4,12 | 4,38E-04 | 6,21E-02 | 0,07 | BCL3 | 602 |
| ILMN_1747207 | 0,65 | 9,94 | 4,39 | 2,25E-04 | 4,81E-02 | 0,67 | SERPINH1 | 871 |
| ILMN_1806757 | 0,65 | 9,09 | 3,72 | 1,17E-03 | 8,74E-02 | -0,81 | EIF5A2 | 56648 |
| ILMN_2411282 | 0,66 | 8,78 | 4,01 | 5,77E-04 | 6,77E-02 | -0,17 | CCDC71L | 168455 |
| ILMN_2107991 | 0,66 | 8,73 | 3,69 | 1,24E-03 | 8,90E-02 | -0,86 | EML2 | 24139 |
| ILMN_2173919 | 0,66 | 10,06 | 6,06 | 3,89E-06 | 1,49E-02 | 4,28 | SHC1 | 6464 |
| ILMN_2357542 | 0,67 | 9,09 | 4,04 | 5,33E-04 | 6,62E-02 | -0,10 | BACH1 | 571 |
| ILMN_1679134 | 0,67 | 11,78 | 3,88 | 7,90E-04 | 7,44E-02 | -0,46 | LGALS1 | 3956 |
| ILMN_1688435 | 0,67 | 7,36 | 4,35 | 2,49E-04 | 4,91E-02 | 0,58 | C2orf27A | 29798 |
| ILMN_1812474 | 0,67 | 11,58 | 4,47 | 1,84E-04 | 4,61E-02 | 0,85 | SERPINF1 | 5176 |
| ILMN_1774350 | 0,69 | 7,17 | 4,54 | 1,53E-04 | 4,57E-02 | 1,02 | ABCA12 | 26154 |
| ILMN_1792384 | 0,69 | 7,58 | 3,96 | 6,49E-04 | 6,97E-02 | -0,28 | PIK3R1 | 5295 |
| ILMN_2341363 | 0,69 | 8,50 | 4,94 | 5,80E-05 | 3,28E-02 | 1,88 | MIR100HG | 399959 |
| ILMN_1758378 | 0,71 | 8,54 | 3,68 | 1,28E-03 | 9,05E-02 | -0,88 | RCC1 | 1104 |
| ILMN_1793919 | 0,71 | 10,89 | 4,19 | 3,66E-04 | 5,71E-02 | 0,24 | RBCK1 | 10616 |
| ILMN_1669366 | 0,72 | 9,15 | 4,45 | 1,92E-04 | 4,61E-02 | 0,82 | RNF128 | 79589 |
| ILMN_2067520 | 0,73 | 7,00 | 3,79 | 9,79E-04 | 8,19E-02 | -0,65 | LBP | 3929 |
| ILMN_1654289 | 0,73 | 7,08 | 5,02 | 4,73E-05 | 3,16E-02 | 2,07 | SLC52A1 | 55065 |
| ILMN_1711909 | 0,75 | 8,71 | 3,73 | 1,14E-03 | 8,69E-02 | -0,78 | RCE1 | 9986 |
| ILMN_1744118 | 0,76 | 10,62 | 3,86 | 8,14E-04 | 7,57E-02 | -0,48 | MRC2 | 9902 |
| ILMN_1729175 | 0,77 | 7,68 | 4,75 | 9,17E-05 | 3,76E-02 | 1,48 | ADAM12 | 8038 |
| ILMN_2179717 | 0,78 | 7,10 | 4,04 | 5,34E-04 | 6,62E-02 | -0,10 | MMP3 | 4314 |
| ILMN_1778478 | 0,79 | 8,19 | 5,25 | 2,73E-05 | 2,69E-02 | 2,56 | TANC2 | 26115 |
| ILMN_1779778 | 0,79 | 7,38 | 3,95 | 6,55E-04 | 7,01E-02 | -0,29 | PLXNB3 | 5365 |
| ILMN_1763539 | 0,80 | 10,28 | 3,79 | 9,69E-04 | 8,19E-02 | -0,64 | SPSB1 | 80176 |
| ILMN_1740430 | 0,80 | 11,18 | 4,37 | 2,35E-04 | 4,81E-02 | 0,63 | PLOD2 | 5352 |
| ILMN_1749410 | 0,80 | 9,15 | 5,30 | 2,38E-05 | 2,49E-02 | 2,68 | PIM1 | 5292 |
| ILMN_1735906 | 0,81 | 8,29 | 5,12 | 3,74E-05 | 2,72E-02 | 2,27 | UHRF1 | 29128 |
| ILMN_1764723 | 0,82 | 10,85 | 4,00 | 5,77E-04 | 6,77E-02 | -0,17 | TGM2 | 7052 |
| ILMN_1802027 | 0,82 | 9,61 | 4,13 | 4,25E-04 | 6,13E-02 | 0,10 | CREB3L1 | 90993 |
| ILMN_1745499 | 0,82 | 8,08 | 3,78 | 9,96E-04 | 8,21E-02 | -0,66 | PLAUR | 5329 |
| ILMN_3300353 | 0,84 | 7,75 | 4,00 | 5,81E-04 | 6,78E-02 | -0,18 | CCDC74B | 91409 |
| ILMN_2328433 | 0,84 | 10,85 | 4,02 | 5,62E-04 | 6,73E-02 | -0,15 | VCAN | 1462 |
| ILMN_1810037 | 0,86 | 7,61 | 4,37 | 2,34E-04 | 4,81E-02 | 0,64 | PLTP | 5360 |
| ILMN_1682935 | 0,86 | 7,69 | 3,89 | 7,67E-04 | 7,34E-02 | -0,43 | ROR2 | 4920 |
| ILMN_1696066 | 0,87 | 10,50 | 3,61 | 1,51E-03 | 9,78E-02 | -1,03 | COL6A3 | 1293 |
| ILMN_2357419 | 0,87 | 7,41 | 3,78 | 9,96E-04 | 8,21E-02 | -0,66 | ADAM12 | 8038 |
| ILMN_1797554 | 0,87 | 11,89 | 4,06 | 5,06E-04 | 6,51E-02 | -0,06 | MT1X | 4501 |
| ILMN_1773470 | 0,88 | 7,34 | 4,75 | 9,10E-05 | 3,76E-02 | 1,48 | NCAM1 | 4684 |
| ILMN_1789507 | 0,88 | 7,28 | 4,36 | 2,40E-04 | 4,81E-02 | 0,61 | WFDC10B | 280664 |
| ILMN_2323633 | 0,88 | 7,90 | 5,48 | 1,55E-05 | 2,15E-02 | 3,06 | TNNI2 | 7136 |
| ILMN_1800634 | 0,89 | 8,34 | 5,22 | 2,89E-05 | 2,69E-02 | 2,51 | LAGE3 | 8270 |
| ILMN_1711210 | 0,89 | 7,41 | 4,69 | 1,07E-04 | 3,93E-02 | 1,34 | COL11A1 | 1301 |
| ILMN_1657475 | 0,90 | 11,26 | 5,12 | 3,68E-05 | 2,72E-02 | 2,29 | NAMPT | 10135 |
| ILMN_2343278 | 0,91 | 7,90 | 3,93 | 6,94E-04 | 7,15E-02 | -0,34 | CXCL1 | 2919 |
| ILMN_1661170 | 0,91 | 8,23 | 4,18 | 3,78E-04 | 5,74E-02 | 0,21 | HIST1H2AH | 85235 |
| ILMN_2342841 | 0,91 | 9,71 | 4,02 | 5,63E-04 | 6,73E-02 | -0,15 | CHPF | 79586 |
| ILMN_1780698 | 0,92 | 9,15 | 4,29 | 2,83E-04 | 5,10E-02 | 0,47 | SHC1 | 6464 |
| ILMN_2328029 | 0,92 | 10,75 | 6,92 | 5,42E-07 | 5,03E-03 | 5,99 | FHL2 | 2274 |
| ILMN_1727249 | 0,92 | 12,25 | 6,52 | 1,33E-06 | 9,14E-03 | 5,21 | MT2A | 4502 |
| ILMN_2128967 | 0,93 | 7,98 | 5,88 | 5,91E-06 | 1,70E-02 | 3,91 | COPZ2 | 51226 |
| ILMN_1728083 | 0,93 | 8,32 | 4,45 | 1,95E-04 | 4,61E-02 | 0,80 | FKBP10 | 60681 |
| ILMN_1748090 | 0,93 | 7,50 | 3,75 | 1,07E-03 | 8,55E-02 | -0,72 | FCHO1 | 23149 |
| ILMN_1674975 | 0,95 | 8,72 | 3,74 | 1,09E-03 | 8,55E-02 | -0,74 | C1S | 716 |
| ILMN_2304404 | 0,95 | 8,92 | 4,31 | 2,70E-04 | 5,01E-02 | 0,51 | EIF4EBP1 | 1978 |
| ILMN_1710408 | 0,95 | 8,49 | 3,75 | 1,08E-03 | 8,55E-02 | -0,74 | TK1 | 7083 |
| ILMN_2046003 | 0,95 | 9,85 | 5,58 | 1,23E-05 | 2,09E-02 | 3,26 | SEMA4B | 10509 |
| ILMN_1806106 | 0,96 | 9,91 | 4,56 | 1,48E-04 | 4,49E-02 | 1,05 | STK39 | 27347 |
| ILMN_2158336 | 0,96 | 11,70 | 4,18 | 3,75E-04 | 5,74E-02 | 0,21 | THBS2 | 7058 |
| ILMN_1767992 | 0,96 | 9,84 | 4,60 | 1,32E-04 | 4,47E-02 | 1,15 | NAMPT | 10135 |
| ILMN_1808163 | 0,97 | 7,76 | 4,76 | 8,99E-05 | 3,76E-02 | 1,49 | SPOCD1 | 90853 |
| ILMN_1665192 | 0,97 | 8,73 | 4,11 | 4,41E-04 | 6,21E-02 | 0,07 | ADAMTS2 | 9509 |
| ILMN_1687303 | 0,98 | 11,45 | 3,74 | 1,11E-03 | 8,60E-02 | -0,76 | SLC7A5 | 8140 |
| ILMN_1693878 | 0,98 | 8,82 | 3,89 | 7,63E-04 | 7,34E-02 | -0,42 | MANEAL | 149175 |
| ILMN_1749218 | 0,99 | 9,15 | 4,12 | 4,31E-04 | 6,17E-02 | 0,09 | TGM2 | 7052 |
| ILMN_2367469 | 0,99 | 7,08 | 4,94 | 5,77E-05 | 3,28E-02 | 1,89 | CXCL5 | 6374 |
| ILMN_1788783 | 0,99 | 8,20 | 3,91 | 7,35E-04 | 7,32E-02 | -0,39 | RARRES1 | 5918 |
| ILMN_1669747 | 0,99 | 8,63 | 3,85 | 8,48E-04 | 7,72E-02 | -0,52 | KDELR3 | 11015 |
| ILMN_1683107 | 1,02 | 10,94 | 4,63 | 1,22E-04 | 4,26E-02 | 1,22 | SMIM3 | 85027 |
| ILMN_2127328 | 1,02 | 7,72 | 3,71 | 1,18E-03 | 8,74E-02 | -0,81 | NA | 81557 |
| ILMN_1736654 | 1,02 | 7,24 | 4,01 | 5,66E-04 | 6,73E-02 | -0,16 | PI3 | 5266 |
| ILMN_1659888 | 1,04 | 8,31 | 3,95 | 6,59E-04 | 7,04E-02 | -0,29 | FAP | 2191 |
| ILMN_2396672 | 1,04 | 7,91 | 3,74 | 1,10E-03 | 8,56E-02 | -0,75 | CHRDL2 | 25884 |
| ILMN_1665049 | 1,05 | 11,19 | 3,97 | 6,34E-04 | 6,97E-02 | -0,26 | COL1A1 | 1277 |
| ILMN_1793854 | 1,07 | 8,86 | 4,45 | 1,91E-04 | 4,61E-02 | 0,82 | COL5A1 | 1289 |
| ILMN_1690291 | 1,07 | 10,61 | 4,51 | 1,67E-04 | 4,57E-02 | 0,94 | C1R | 715 |
| ILMN_2167805 | 1,08 | 7,49 | 3,60 | 1,55E-03 | 9,93E-02 | -1,06 | PRAME | 23532 |
| ILMN_1732720 | 1,08 | 9,17 | 4,03 | 5,37E-04 | 6,64E-02 | -0,11 | SHC1 | 6464 |
| ILMN_1800721 | 1,09 | 8,18 | 4,21 | 3,52E-04 | 5,64E-02 | 0,27 | FNDC1 | 84624 |
| ILMN_1660021 | 1,09 | 8,06 | 4,31 | 2,69E-04 | 5,01E-02 | 0,51 | FADS2 | 9415 |
| ILMN_1707339 | 1,10 | 8,20 | 3,92 | 7,13E-04 | 7,26E-02 | -0,36 | MLLT11 | 10962 |
| ILMN_1792135 | 1,10 | 9,90 | 3,81 | 9,42E-04 | 8,11E-02 | -0,61 | CXCL2 | 2920 |
| ILMN_1692684 | 1,11 | 9,19 | 3,70 | 1,21E-03 | 8,77E-02 | -0,83 | FOSL1 | 8061 |
| ILMN_1656386 | 1,11 | 9,56 | 3,69 | 1,26E-03 | 8,99E-02 | -0,87 | PLAUR | 5329 |
| ILMN_2412849 | 1,11 | 10,51 | 4,56 | 1,46E-04 | 4,49E-02 | 1,06 | SFRP4 | 6424 |
| ILMN_3253456 | 1,12 | 9,22 | 5,18 | 3,20E-05 | 2,72E-02 | 2,41 | FHL2 | 2274 |
| ILMN_1719518 | 1,12 | 7,38 | 5,47 | 1,58E-05 | 2,15E-02 | 3,04 | NCAM1 | 4684 |
| ILMN_2394264 | 1,14 | 7,90 | 4,29 | 2,88E-04 | 5,11E-02 | 0,45 | WFDC3 | 140686 |
| ILMN_1810488 | 1,15 | 7,92 | 4,04 | 5,26E-04 | 6,62E-02 | -0,09 | UBE2C | 11065 |
| ILMN_1741960 | 1,17 | 8,04 | 3,86 | 8,19E-04 | 7,59E-02 | -0,49 | MARCO | 8685 |
| ILMN_2358134 | 1,17 | 9,29 | 3,70 | 1,20E-03 | 8,77E-02 | -0,83 | DAND5 | 199699 |
| ILMN_1675646 | 1,18 | 8,85 | 4,14 | 4,12E-04 | 6,10E-02 | 0,13 | SLC7A5P1 | 8140 |
| ILMN_1672776 | 1,19 | 8,60 | 4,31 | 2,75E-04 | 5,01E-02 | 0,49 | KRTCAP3 | 200634 |
| ILMN_1655642 | 1,19 | 8,27 | 7,43 | 1,73E-07 | 2,99E-03 | 6,96 | SLC2A14 | 144195 |
| ILMN_1748189 | 1,20 | 9,90 | 3,96 | 6,48E-04 | 6,97E-02 | -0,28 | CFH | 3075 |
| ILMN_1782705 | 1,21 | 8,70 | 4,08 | 4,84E-04 | 6,42E-02 | -0,02 | POSTN | 10631 |
| ILMN_1733419 | 1,23 | 9,74 | 3,79 | 9,74E-04 | 8,19E-02 | -0,64 | POSTN | 10631 |
| ILMN_2352303 | 1,23 | 9,25 | 4,74 | 9,33E-05 | 3,76E-02 | 1,46 | SERPINE1 | 5054 |
| ILMN_2083833 | 1,25 | 9,17 | 4,11 | 4,41E-04 | 6,21E-02 | 0,07 | ADA | 100 |
| ILMN_2361400 | 1,29 | 8,38 | 4,51 | 1,68E-04 | 4,57E-02 | 0,93 | IL1R2 | 7850 |
| ILMN_1766650 | 1,31 | 10,84 | 4,52 | 1,64E-04 | 4,57E-02 | 0,96 | LIF | 3976 |
| ILMN_1683766 | 1,31 | 9,58 | 4,99 | 5,14E-05 | 3,16E-02 | 1,99 | SPOCK1 | 6695 |
| ILMN_2345837 | 1,33 | 8,53 | 4,13 | 4,20E-04 | 6,13E-02 | 0,11 | GXYLT2 | 727936 |
| ILMN_1679958 | 1,36 | 8,21 | 5,41 | 1,85E-05 | 2,20E-02 | 2,90 | GFPT2 | 9945 |
| ILMN_2358382 | 1,37 | 8,64 | 5,75 | 8,10E-06 | 2,09E-02 | 3,63 | CYP1B1 | 1545 |
| ILMN_1750497 | 1,38 | 9,93 | 3,75 | 1,08E-03 | 8,55E-02 | -0,74 | RARRES1 | 5918 |
| ILMN_1805652 | 1,40 | 9,62 | 4,36 | 2,39E-04 | 4,81E-02 | 0,62 | PLTP | 5360 |
| ILMN_3181457 | 1,42 | 7,71 | 3,97 | 6,26E-04 | 6,97E-02 | -0,25 | B3GALT5 | 10317 |
| ILMN_1787556 | 1,42 | 8,97 | 4,38 | 2,30E-04 | 4,81E-02 | 0,65 | CTHRC1 | 115908 |
| ILMN_1660440 | 1,43 | 9,44 | 3,81 | 9,25E-04 | 8,04E-02 | -0,60 | PDGFRL | 5157 |
| ILMN_1664630 | 1,45 | 9,57 | 4,36 | 2,40E-04 | 4,81E-02 | 0,61 | EFNA5 | 1946 |
| ILMN_1677942 | 1,46 | 7,99 | 6,09 | 3,65E-06 | 1,49E-02 | 4,33 | APLP1 | 333 |
| ILMN_3243578 | 1,47 | 8,48 | 5,13 | 3,60E-05 | 2,72E-02 | 2,31 | TRIB3 | 57761 |
| ILMN_1812226 | 1,48 | 7,76 | 4,60 | 1,34E-04 | 4,47E-02 | 1,14 | AQP9 | 366 |
| ILMN_1781814 | 1,50 | 7,89 | 4,56 | 1,48E-04 | 4,49E-02 | 1,04 | ITPKA | 3706 |
| ILMN_2250820 | 1,52 | 7,62 | 4,33 | 2,61E-04 | 4,97E-02 | 0,54 | EYA1 | 2138 |
| ILMN_1701244 | 1,53 | 10,52 | 4,49 | 1,73E-04 | 4,61E-02 | 0,91 | LOX | 4015 |
| ILMN_1739914 | 1,55 | 8,00 | 3,99 | 5,99E-04 | 6,89E-02 | -0,21 | CCL20 | 6364 |
| ILMN_2295290 | 1,56 | 7,95 | 4,45 | 1,92E-04 | 4,61E-02 | 0,81 | STEAP3 | 55240 |
| ILMN_1697448 | 1,56 | 9,68 | 4,59 | 1,36E-04 | 4,47E-02 | 1,12 | GPRC5A | 9052 |
| ILMN_1802524 | 1,57 | 8,25 | 3,86 | 8,33E-04 | 7,64E-02 | -0,50 | PITX2 | 5308 |
| ILMN_2284400 | 1,60 | 7,74 | 5,46 | 1,62E-05 | 2,15E-02 | 3,02 | IGF2BP3 | 10643 |
| ILMN_2242345 | 1,64 | 8,09 | 4,01 | 5,70E-04 | 6,76E-02 | -0,16 | CXCL8 | 3576 |
| ILMN_1677693 | 1,65 | 9,94 | 4,50 | 1,68E-04 | 4,57E-02 | 0,93 | TUBB3 | 10381 |
| ILMN_1680513 | 1,71 | 9,48 | 3,80 | 9,50E-04 | 8,11E-02 | -0,62 | PRPH | 5630 |
| ILMN_2098119 | 1,78 | 10,01 | 4,31 | 2,71E-04 | 5,01E-02 | 0,50 | IL6 | 3569 |
| ILMN_2072045 | 1,78 | 8,54 | 4,69 | 1,07E-04 | 3,93E-02 | 1,33 | MAP7D2 | 256714 |
| ILMN_1762741 | 1,81 | 8,33 | 3,67 | 1,30E-03 | 9,09E-02 | -0,90 | PTHLH | 5744 |
| ILMN_1685493 | 1,87 | 8,65 | 5,71 | 8,99E-06 | 2,09E-02 | 3,54 | COMP | 1311 |
| ILMN_2313434 | 1,90 | 8,96 | 5,16 | 3,37E-05 | 2,72E-02 | 2,37 | STEAP3 | 55240 |
| ILMN_1679898 | 1,96 | 7,64 | 4,40 | 2,16E-04 | 4,79E-02 | 0,71 | IGFBP1 | 3484 |
| ILMN_1720300 | 2,03 | 9,15 | 4,18 | 3,78E-04 | 5,74E-02 | 0,21 | NCAM1 | 4684 |
| ILMN_1703229 | 2,06 | 9,00 | 4,23 | 3,33E-04 | 5,49E-02 | 0,32 | SERPINA3 | 12 |
| ILMN_3251264 | 2,07 | 9,65 | 4,68 | 1,09E-04 | 3,95E-02 | 1,32 | TMEM145 | 284339 |
| ILMN_1846110 | 2,21 | 9,45 | 3,84 | 8,58E-04 | 7,73E-02 | -0,53 | PLA2G2A | 5320 |
| ILMN_1676624 | 2,23 | 9,71 | 3,80 | 9,47E-04 | 8,11E-02 | -0,62 | MMP9 | 4318 |
| ILMN_1709717 | 2,28 | 7,95 | 4,19 | 3,70E-04 | 5,71E-02 | 0,23 | GNAS | 2778 |
