# Supplementary material for: Patterns of gene expression characterize T1 and T3 clear cell renal cell carcinoma subtypes
Source: PLoS One. 2019 May 31;14(5):e0216793. doi: 10.1371/journal.pone.0216793 (PMC6544217; doi:10.1371/journal.pone.0216793)
Supplement: S3 Table — All probes that reached adj. p. value < 0.05 cut-off value. ILMN ID–Illumina probe ID, logFC–log Fold Change, AveExpr–average probe expression value, P.Value–p value, adj.P.Val–p value adjusted for multiple testing. (DOCX) [file pone.0216793.s003.docx]

**S3 Table** List of differentially expressed genes in A2 vs A1 and A2 vs A3 comparisons. All probes that reached adj. p. value < 0.05 cut-off value. ILMN ID – Illumina probe ID, logFC – log Fold Change,

AveExpr – average probe expression value, P.Value – p value, adj.P.Val – p value adjusted for multiple testing.

| A2 vs A1 | ILMN ID | logFC | AveExpr | P.Value | adj.P.Val | Gene ID | Associated Gene Name | Entrez |
| --- | --- | --- | --- | --- | --- | --- | --- | --- |
|  | ILMN_1677942 | -0,30 | 7,07 | 1,01E-05 | 4,16E-02 | ENSG00000198892 | SHISA4 | 149345 |
|  | ILMN_1679232 | 0,39 | 10,12 | 1,9E-05 | 4,89E-02 | ENSG00000134313 | KIDINS220 | 57498 |
|  | ILMN_1681583 | -0,88 | 9,09 | 3,22E-06 | 4,16E-02 | ENSG00000156273 | BACH1 | 571 |
|  | ILMN_1698725 | 1,11 | 8,58 | 1,21E-05 | 4,16E-02 | ENSG00000172159 | FRMD3 | 257019 |
|  | ILMN_1699651 | -1,99 | 10,01 | 1,12E-05 | 4,16E-02 | ENSG00000136244 | IL6 | 3569 |
|  | ILMN_1712719 | 1,03 | 8,19 | 1,19E-05 | 4,16E-02 | ENSG00000135525 | MAP7 | 9053 |
|  | ILMN_1731157 | 1,13 | 9,25 | 2,13E-05 | 4,89E-02 | ENSG00000177791 | MYOZ1 | 58529 |
|  | ILMN_1764109 | -1,24 | 10,61 | 1,09E-05 | 4,16E-02 | ENSG00000159403 | C1R | 715 |
|  | ILMN_1784523 | 0,84 | 9,90 | 1,33E-05 | 4,16E-02 | ENSG00000136888 | ATP6V1G1 | 9550 |
|  | ILMN_1810037 | 0,60 | 7,98 | 1,32E-06 | 4,16E-02 | ENSG00000198853 | RUSC2 | 9853 |
|  | ILMN_2290338 | -0,96 | 7,98 | 2,11E-05 | 4,89E-02 | ENSG00000159200 | RCAN1 | 1827 |
|  | ILMN_2388800 | 0,79 | 9,69 | 1,9E-05 | 4,89E-02 | ENSG00000162407 | PLPP3 | 8613 |
|  | ILMN_2408683 | 0,76 | 8,44 | 7,45E-06 | 4,16E-02 | ENSG00000162407 | PLPP3 | 8613 |
|  | ILMN_3237679 | 0,82 | 9,13 | 5,32E-06 | 4,16E-02 | ENSG00000188647 | PTAR1 | 375743 |
|  | ILMN_3251477 | 0,51 | 7,88 | 1,05E-05 | 4,16E-02 | ENSG00000165275 | TRMT10B | 158234 |
| A2 vs A3 | ILMN ID | logFC | AveExpr | P.Value | adj.P.Val | Gene ID | Associated Gene Name | Entrez |
|  | ILMN_2089977 | -1,99 | 8,99 | 5,94E-06 | 1,09E-02 | ENSG00000176826 | FKBP9P1 | 360132 |
|  | ILMN_1807423 | -1,65 | 7,74 | 4,13E-06 | 1,02E-02 | ENSG00000136231 | IGF2BP3 | 10643 |
|  | ILMN_2171384 | -1,11 | 7,08 | 3,32E-05 | 3,70E-02 | ENSG00000163735 | CXCL5 | 6374 |
|  | ILMN_1741465 | -0,80 | 6,99 | 3,53E-06 | 9,36E-03 | ENSG00000080031 | PTPRH | 5794 |
|  | ILMN_1758034 | 0,69 | 11,08 | 1,14E-06 | 6,16E-03 | ENSG00000171503 | ETFDH | 2110 |
|  | ILMN_3239378 | 0,72 | 10,38 | 1,85E-05 | 2,55E-02 | ENSG00000173011 | TADA2B | 93624 |
|  | ILMN_1694535 | 0,81 | 7,75 | 2,41E-05 | 2,97E-02 | ENSG00000174827 | PDZK1 | 5174 |
|  | ILMN_2144401 | 0,92 | 7,94 | 3,33E-05 | 3,70E-02 | ENSG00000149328 | GLB1L2 | 89944 |
|  | ILMN_2096372 | 0,95 | 11,63 | 1,37E-05 | 1,98E-02 | ENSG00000165092 | ALDH1A1 | 216 |
|  | ILMN_1690327 | 0,96 | 10,06 | 1,96E-06 | 6,16E-03 | ENSG00000171365 | CLCN5 | 1184 |
|  | ILMN_1734365 | 1,04 | 7,75 | 2,12E-05 | 2,70E-02 | ENSG00000075891 | PAX2 | 5076 |
|  | ILMN_2231911 | 1,10 | 10,53 | 5,68E-06 | 1,09E-02 | ENSG00000148090 | AUH | 549 |
|  | ILMN_1748323 | 1,16 | 11,32 | 2,73E-05 | 3,24E-02 | ENSG00000145824 | CXCL14 | 9547 |
|  | ILMN_1691503 | 1,61 | 8,38 | 5,67E-06 | 1,09E-02 | ENSG00000154025 | SLC5A10 | 125206 |
|  | ILMN_1665033 | 1,62 | 9,07 | 1,21E-05 | 1,89E-02 | ENSG00000113389 | NPR3 | 4883 |
|  | ILMN_1728009 | 1,71 | 8,76 | 1,38E-05 | 1,98E-02 | ENSG00000157111 | TMEM171 | 134285 |
|  | ILMN_1751232 | 1,76 | 8,62 | 1,81E-06 | 6,16E-03 | ENSG00000154025 | SLC5A10 | 125206 |
|  | ILMN_1664350 | 1,76 | 9,37 | 1,04E-05 | 1,71E-02 | ENSG00000249948 | GBA3 | 57733 |
|  | ILMN_2223313 | 1,80 | 8,61 | 5,42E-06 | 1,09E-02 | ENSG00000205795 | CYS1 | 192668 |
|  | ILMN_2181064 | 2,06 | 9,90 | 1,96E-05 | 2,60E-02 | ENSG00000249948 | GBA3 | 57733 |
|  | ILMN_1772894 | 2,30 | 10,01 | 4,8E-07 | 6,10E-03 | ENSG00000147003 | TMEM27 | 57393 |
|  | ILMN_2353490 | 2,31 | 10,53 | 5,56E-07 | 6,10E-03 | ENSG00000075891 | PAX2 | 5076 |
|  | ILMN_1740402 | 2,33 | 8,85 | 1,54E-06 | 6,16E-03 | ENSG00000116882 | HAO2 | 51179 |
|  | ILMN_1765912 | 2,43 | 9,68 | 6,01E-06 | 1,09E-02 | ENSG00000066813 | ACSM2B | 348158 |
|  | ILMN_1746128 | 2,49 | 10,12 | 6,76E-06 | 1,16E-02 | ENSG00000066813 | ACSM2B | 348158 |
|  | ILMN_2334193 | 2,59 | 9,46 | 4,47E-08 | 1,54E-03 | ENSG00000112499 | SLC22A2 | 6582 |
|  | ILMN_1767474 | 2,67 | 9,57 | 1,76E-06 | 6,16E-03 | ENSG00000116882 | HAO2 | 51179 |
|  | ILMN_1762410 | 2,85 | 10,11 | 7,08E-07 | 6,10E-03 | ENSG00000112499 | SLC22A2 | 6582 |
|  | ILMN_1815480 | 2,90 | 11,08 | 1,66E-06 | 6,16E-03 | ENSG00000144035 | NAT8 | 9027 |
|  | ILMN_1731433 | 2,99 | 11,43 | 2,92E-06 | 8,38E-03 | ENSG00000002726 | AOC1 | 26 |
|  | ILMN_2228463 | 3,02 | 10,82 | 1,23E-06 | 6,16E-03 | ENSG00000112499 | SLC22A2 | 6582 |
