# Supplementary material for: Patterns of gene expression characterize T1 and T3 clear cell renal cell carcinoma subtypes
Source: PLoS One. 2019 May 31;14(5):e0216793. doi: 10.1371/journal.pone.0216793 (PMC6544217; doi:10.1371/journal.pone.0216793)
Supplement: S4 Table — All probes that reached adj. p. value < 0.05 and logFC > 1.5 cut-off values. ILMN ID–Illumina probe ID, logFC–log Fold Change, AveExpr–average probe expression value, P.Value–p value, adj.P.Val–p value adjusted for multiple testing. (DOCX) [file pone.0216793.s004.docx]

**S4 Table** List of differentially expressed genes in A3 vs A1 comparison. All probes that reached adj. p. value < 0.05 and logFC > 1.5 cut-off values. ILMN ID – Illumina probe ID, logFC – log Fold Change, AveExpr – average probe expression value, P.Value – p value, adj.P.Val – p value adjusted for multiple

testing.

| ILMN ID | logFC | AveExpr | P.Value | adj.P.Val | Gene symbol | Entrez |
| --- | --- | --- | --- | --- | --- | --- |
| ILMN_2334193 | 3,19 | 9,46 | 8,15E-11 | 2,13E-06 | SLC22A2 | 6582 |
| ILMN_1665033 | 2,79 | 9,07 | 1,51E-10 | 2,13E-06 | NPR3 | 4883 |
| ILMN_1751232 | 2,62 | 8,62 | 1,86E-10 | 2,13E-06 | SLC5A10 | 125206 |
| ILMN_1772894 | 3,01 | 10,01 | 4,21E-10 | 3,63E-06 | TMEM27 | 57393 |
| ILMN_2181064 | 3,41 | 9,90 | 6,76E-10 | 4,55E-06 | GBA3 | 57733 |
| ILMN_1664350 | 2,75 | 9,37 | 7,91E-10 | 4,55E-06 | GBA3 | 57733 |
| ILMN_1767474 | 3,62 | 9,57 | 1,04E-09 | 5,13E-06 | HAO2 | 51179 |
| ILMN_1691503 | 2,29 | 8,38 | 1,83E-09 | 7,78E-06 | SLC5A10 | 125206 |
| ILMN_1807423 | -2,29 | 7,74 | 2,12E-09 | 7,78E-06 | IGF2BP3 | 10643 |
| ILMN_1762410 | 3,44 | 10,11 | 2,93E-09 | 9,18E-06 | SLC22A2 | 6582 |
| ILMN_1681103 | 2,07 | 10,01 | 1,02E-08 | 2,32E-05 | AQP1 | 358 |
| ILMN_2353490 | 2,55 | 10,53 | 1,08E-08 | 2,32E-05 | PAX2 | 5076 |
| ILMN_1740402 | 2,72 | 8,85 | 1,31E-08 | 2,66E-05 | HAO2 | 51179 |
| ILMN_2228463 | 3,32 | 10,82 | 2,93E-08 | 5,6E-05 | DDC | 1644 |
| ILMN_1766083 | 3,86 | 9,10 | 3,53E-08 | 6,23E-05 | SLC22A12 | 116085 |
| ILMN_1729180 | 2,08 | 10,30 | 3,61E-08 | 6,23E-05 | GATM | 2628 |
| ILMN_1712719 | 1,58 | 8,19 | 3,9E-08 | 6,31E-05 | MAP7 | 9053 |
| ILMN_1731433 | 3,41 | 11,43 | 4,03E-08 | 6,31E-05 | AOC1 | 26 |
| ILMN_2302983 | 2,93 | 9,90 | 5,42E-08 | 8,12E-05 | TRPM3 | 80036 |
| ILMN_1765912 | 2,85 | 9,68 | 6,26E-08 | 8,63E-05 | ACSM2B | 348158 |
| ILMN_1815480 | 3,02 | 11,08 | 1E-07 | 0,000104 | NAT8 | 9027 |
| ILMN_1722829 | 1,71 | 9,48 | 1,01E-07 | 0,000104 | HLF | 3131 |
| ILMN_1746128 | 2,86 | 10,12 | 1,02E-07 | 0,000104 | ACSM2B | 348158 |
| ILMN_1667018 | 2,19 | 8,09 | 1,21E-07 | 0,000109 | ACE2 | 59272 |
| ILMN_1806710 | 2,04 | 11,37 | 2,2E-07 | 0,000171 | ESPN | 83715 |
| ILMN_1728009 | 1,97 | 8,76 | 2,23E-07 | 0,000171 | TMEM171 | 134285 |
| ILMN_2187746 | 2,11 | 8,88 | 2,32E-07 | 0,000174 | EMX2 | 2018 |
| ILMN_2216815 | 1,64 | 8,58 | 3,05E-07 | 0,000191 | MAP7 | 9053 |
| ILMN_2223313 | 1,89 | 8,61 | 3,34E-07 | 0,000198 | CYS1 | 192668 |
| ILMN_1674353 | 3,42 | 10,07 | 4,84E-07 | 0,000269 | MIOX | 55586 |
| ILMN_1720034 | 2,18 | 8,64 | 5,08E-07 | 0,000278 | PKHD1 | 5314 |
| ILMN_2412192 | -1,82 | 9,90 | 5,72E-07 | 0,000308 | CFH | 3075 |
| ILMN_1809291 | 1,62 | 9,04 | 6,32E-07 | 0,00033 | TSPAN7 | 7102 |
| ILMN_1798992 | 2,03 | 10,86 | 7E-07 | 0,000355 | MYL3 | 4634 |
| ILMN_1693338 | -1,70 | 8,64 | 7,44E-07 | 0,000361 | CYP1B1 | 1545 |
| ILMN_1690979 | 2,58 | 9,09 | 8,29E-07 | 0,000397 | SLC17A3 | 10786 |
| ILMN_1810172 | -1,62 | 10,51 | 9,08E-07 | 0,000429 | SFRP4 | 6424 |
| ILMN_2196328 | -1,77 | 8,70 | 9,67E-07 | 0,00045 | POSTN | 10631 |
| ILMN_1750062 | 2,71 | 9,90 | 1,16E-06 | 0,000514 | PPARGC1A | 10891 |
| ILMN_2308903 | -1,64 | 7,90 | 1,26E-06 | 0,000519 | WFDC3 | 140686 |
| ILMN_1713561 | -2,18 | 8,14 | 1,39E-06 | 0,000563 | LAMP5 | 24141 |
| ILMN_1803686 | -1,83 | 9,17 | 1,44E-06 | 0,00057 | ADA | 100 |
| ILMN_1791726 | -2,29 | 9,94 | 1,49E-06 | 0,000571 | TUBB3 | 10381 |
| ILMN_1803197 | 1,68 | 9,27 | 1,65E-06 | 0,000617 | RAB3IP | 117177 |
| ILMN_1723443 | 2,12 | 11,54 | 1,66E-06 | 0,000617 | LRP2 | 4036 |
| ILMN_2216637 | 1,61 | 9,98 | 1,9E-06 | 0,000696 | STK32B | 55351 |
| ILMN_2145396 | 1,77 | 8,50 | 1,97E-06 | 0,000704 | AKR7A3 | 22977 |
| ILMN_1701507 | 1,51 | 9,91 | 2,19E-06 | 0,000754 | EHHADH | 1962 |
| ILMN_1663569 | 2,93 | 10,17 | 2,24E-06 | 0,000765 | FTCD | 10841 |
| ILMN_2301083 | -1,70 | 7,92 | 2,31E-06 | 0,00078 | UBE2C | 11065 |
| ILMN_1787815 | -1,87 | 8,48 | 2,63E-06 | 0,000855 | TRIB3 | 57761 |
| ILMN_1707169 | 2,09 | 9,59 | 2,67E-06 | 0,00086 | AGMAT | 79814 |
| ILMN_1734929 | 2,26 | 9,82 | 2,72E-06 | 0,000867 | BBOX1 | 8424 |
| ILMN_1714577 | 2,29 | 9,48 | 3,13E-06 | 0,000961 | OGDHL | 55753 |
| ILMN_1761275 | 1,76 | 8,37 | 3,17E-06 | 0,000961 | FUT6 | 2528 |
| ILMN_1749579 | 2,04 | 8,87 | 3,21E-06 | 0,000961 | PKHD1 | 5314 |
| ILMN_2232854 | -1,53 | 8,31 | 3,56E-06 | 0,001048 | FAP | 2191 |
| ILMN_2089977 | -1,79 | 8,99 | 3,81E-06 | 0,001103 | FKBP9P1 | 360132 |
| ILMN_1740744 | -2,07 | 7,79 | 4,02E-06 | 0,001127 | PRAME | 23532 |
| ILMN_2306033 | -1,65 | 7,49 | 4,43E-06 | 0,001213 | PRAME | 23532 |
| ILMN_1695193 | 3,02 | 8,42 | 4,43E-06 | 0,001213 | ANGPTL3 | 27329 |
| ILMN_1685616 | -1,97 | 9,57 | 4,59E-06 | 0,001236 | EFNA5 | 1946 |
| ILMN_2378654 | -2,07 | 7,71 | 4,81E-06 | 0,001257 | B3GALT5 | 10317 |
| ILMN_1677636 | -2,33 | 8,65 | 5,91E-06 | 0,001499 | COMP | 1311 |
| ILMN_1763834 | -1,72 | 7,99 | 6,05E-06 | 0,001523 | APLP1 | 333 |
| ILMN_1740076 | 1,68 | 8,38 | 6,3E-06 | 0,001541 | SLC16A12 | 387700 |
| ILMN_1790761 | -1,78 | 9,74 | 6,87E-06 | 0,001613 | POSTN | 10631 |
| ILMN_1701308 | -1,50 | 11,19 | 7,48E-06 | 0,001675 | COL1A1 | 1277 |
| ILMN_2120210 | 1,53 | 10,10 | 7,57E-06 | 0,001684 | RCAN2 | 10231 |
| ILMN_1740949 | 1,76 | 7,81 | 7,96E-06 | 0,001759 | LRRC19 | 64922 |
| ILMN_1695157 | 2,64 | 10,69 | 8,64E-06 | 0,001863 | CA4 | 762 |
| ILMN_1794951 | 2,24 | 9,55 | 8,72E-06 | 0,001863 | SLC39A5 | 283375 |
| ILMN_1699651 | -2,12 | 10,01 | 8,92E-06 | 0,001863 | IL6 | 3569 |
| ILMN_1759097 | -1,58 | 8,20 | 1,02E-05 | 0,002059 | MLLT11 | 10962 |
| ILMN_1808157 | 1,76 | 10,36 | 1,26E-05 | 0,002422 | RUNDC3B | 154661 |
| ILMN_2390017 | 3,96 | 9,22 | 1,28E-05 | 0,002427 | SLC22A6 | 9356 |
| ILMN_1807491 | -1,80 | 8,44 | 1,54E-05 | 0,002764 | LAIR2 | 3904 |
| ILMN_1763196 | 2,39 | 11,11 | 1,55E-05 | 0,002764 | WDR72 | 256764 |
| ILMN_2391400 | -2,26 | 8,25 | 1,57E-05 | 0,00278 | PITX2 | 5308 |
| ILMN_2091978 | 1,89 | 8,91 | 1,71E-05 | 0,002914 | FREM2 | 341640 |
| ILMN_2199389 | 1,64 | 11,36 | 1,77E-05 | 0,003003 | VIPR1 | 7433 |
| ILMN_1777031 | 2,95 | 9,73 | 1,88E-05 | 0,003103 | PKLR | 5313 |
| ILMN_1747716 | 4,03 | 9,14 | 1,95E-05 | 0,003159 | ALDOB | 229 |
| ILMN_1789112 | -2,63 | 9,65 | 1,96E-05 | 0,003162 | TMEM145 | 284339 |
| ILMN_1709674 | -1,62 | 8,21 | 2,05E-05 | 0,003262 | GFPT2 | 9945 |
| ILMN_2136971 | 2,01 | 9,01 | 2,17E-05 | 0,003404 | FABP3 | 2170 |
| ILMN_1711748 | -1,78 | 9,62 | 2,36E-05 | 0,003585 | PLTP | 5360 |
| ILMN_2323933 | -1,63 | 7,49 | 2,65E-05 | 0,003942 | LAIR2 | 3904 |
| ILMN_2314169 | -2,67 | 8,33 | 2,68E-05 | 0,003973 | PTHLH | 5744 |
| ILMN_3239643 | 2,90 | 8,79 | 2,9E-05 | 0,004239 | LOC389332 | 389332 |
| ILMN_1770725 | 1,86 | 8,43 | 2,97E-05 | 0,004264 | AIF1L | 83543 |
| ILMN_1725090 | -1,85 | 8,97 | 3,12E-05 | 0,0043 | CTHRC1 | 115908 |
| ILMN_2387385 | -2,56 | 7,64 | 3,16E-05 | 0,0043 | IGFBP1 | 3484 |
| ILMN_2383349 | -2,21 | 8,96 | 3,36E-05 | 0,004471 | STEAP3 | 55240 |
| ILMN_1700031 | -2,28 | 8,05 | 3,5E-05 | 0,004575 | PRAME | 23532 |
| ILMN_1668510 | 1,68 | 8,64 | 3,69E-05 | 0,004724 | GLYAT | 10249 |
| ILMN_1678710 | 2,04 | 9,08 | 3,82E-05 | 0,004866 | PHYHIPL | 84457 |
| ILMN_1810684 | 1,96 | 8,94 | 4,48E-05 | 0,005347 | TINAG | 27283 |
| ILMN_1695579 | 1,78 | 8,90 | 4,67E-05 | 0,005471 | CIT | 11113 |
| ILMN_1796316 | -2,84 | 9,71 | 4,71E-05 | 0,005483 | MMP9 | 4318 |
| ILMN_1761577 | -1,86 | 7,95 | 4,93E-05 | 0,005497 | STEAP3 | 55240 |
| ILMN_1738725 | -1,52 | 10,84 | 4,97E-05 | 0,005497 | LIF | 3976 |
| ILMN_2408748 | 1,54 | 7,52 | 5,15E-05 | 0,005519 | SLC22A12 | 116085 |
| ILMN_1735445 | 1,66 | 9,62 | 5,72E-05 | 0,005885 | SLC7A9 | 11136 |
| ILMN_1679646 | 2,72 | 9,97 | 7,26E-05 | 0,006957 | FTCD | 10841 |
| ILMN_1728799 | 1,90 | 9,41 | 7,6E-05 | 0,0072 | FBP1 | 2203 |
| ILMN_1805665 | 2,06 | 9,09 | 8,05E-05 | 0,007401 | FLRT3 | 23767 |
| ILMN_1696099 | 1,62 | 9,12 | 8,36E-05 | 0,007563 | ALDH4A1 | 8659 |
| ILMN_1740586 | -2,98 | 9,45 | 8,88E-05 | 0,007789 | PLA2G2A | 5320 |
| ILMN_2085844 | -1,66 | 8,54 | 8,97E-05 | 0,007793 | GXYLT2 | 727936 |
| ILMN_1681526 | 2,70 | 9,04 | 9,04E-05 | 0,007827 | SLC5A1 | 6523 |
| ILMN_1692861 | 1,59 | 9,04 | 0,000112 | 0,009026 | UGT2A3 | 79799 |
| ILMN_2405391 | 1,59 | 7,67 | 0,000133 | 0,010225 | SLC22A6 | 9356 |
| ILMN_2080080 | -2,01 | 8,54 | 0,000139 | 0,010439 | MAP7D2 | 256714 |
| ILMN_1785699 | -2,81 | 8,85 | 0,000151 | 0,010939 | PTHLH | 5744 |
| ILMN_1710903 | 1,62 | 10,16 | 0,000159 | 0,011273 | MAPT | 4137 |
| ILMN_3246037 | 1,72 | 8,09 | 0,000163 | 0,011524 | ASPG | 374569 |
| ILMN_1705627 | 1,84 | 9,21 | 0,000163 | 0,011524 | USP2 | 9099 |
| ILMN_1689200 | 2,06 | 8,62 | 0,000173 | 0,011906 | DHDH | 27294 |
| ILMN_2349393 | -1,67 | 8,63 | 0,000175 | 0,012002 | MDK | 4192 |
| ILMN_1755720 | 1,97 | 8,45 | 0,000187 | 0,012452 | SLC2A2 | 6514 |
| ILMN_1686464 | 1,52 | 9,46 | 0,000188 | 0,012455 | SLCO4C1 | 353189 |
| ILMN_1710644 | 1,76 | 8,07 | 0,000203 | 0,013138 | MARVELD3 | 91862 |
| ILMN_1804283 | -2,81 | 7,95 | 0,000205 | 0,013221 | GNAS | 2778 |
| ILMN_1702305 | -2,58 | 9,15 | 0,000212 | 0,013459 | NCAM1 | 4684 |
| ILMN_1743620 | -1,82 | 9,93 | 0,000215 | 0,013545 | RARRES1 | 5918 |
| ILMN_1788874 | -2,54 | 9,00 | 0,000242 | 0,014486 | SERPINA3 | 12 |
| ILMN_1743361 | 2,15 | 9,85 | 0,000242 | 0,014486 | PTGER3 | 5733 |
| ILMN_1680339 | -1,79 | 9,44 | 0,000254 | 0,014925 | PDGFRL | 5157 |
| ILMN_1745299 | -2,02 | 8,01 | 0,000257 | 0,014993 | FABP7 | 2173 |
| ILMN_2296510 | 2,05 | 8,31 | 0,000305 | 0,016672 | SLC5A12 | 159963 |
| ILMN_1682599 | -1,72 | 9,68 | 0,000315 | 0,017032 | GPRC5A | 9052 |
| ILMN_1695880 | -1,76 | 10,52 | 0,00032 | 0,01711 | LOX | 4015 |
| ILMN_1746801 | 2,08 | 9,04 | 0,000322 | 0,017187 | CGN | 57530 |
| ILMN_1758731 | 2,31 | 11,03 | 0,000352 | 0,018163 | CYP2J2 | 1573 |
| ILMN_1754247 | 1,60 | 11,16 | 0,000356 | 0,018262 | SLC3A1 | 6519 |
| ILMN_1715301 | 2,20 | 8,70 | 0,000362 | 0,018459 | FXYD2 | 486 |
| ILMN_1807554 | -1,80 | 7,63 | 0,000384 | 0,019124 | EYA1 | 2138 |
| ILMN_1715068 | -1,59 | 7,76 | 0,000468 | 0,021626 | AQP9 | 366 |
| ILMN_3243185 | 1,61 | 11,00 | 0,000521 | 0,023195 | RERGL | 79785 |
| ILMN_1678669 | -1,62 | 8,77 | 0,000523 | 0,02323 | RRM2 | 6241 |
| ILMN_1734897 | 1,60 | 10,28 | 0,000555 | 0,024055 | SLC4A4 | 8671 |
| ILMN_1784294 | -2,04 | 8,20 | 0,000597 | 0,02502 | CPA4 | 51200 |
| ILMN_1788394 | -2,01 | 8,65 | 0,000621 | 0,025591 | TTLL6 | 284076 |
| ILMN_1681938 | 1,50 | 8,20 | 0,000653 | 0,026194 | ALDH8A1 | 64577 |
| ILMN_1738494 | 1,80 | 10,44 | 0,000714 | 0,02739 | AQP7 | 364 |
| ILMN_1657234 | -1,83 | 8,00 | 0,000717 | 0,0274 | CCL20 | 6364 |
| ILMN_1718173 | 1,64 | 8,19 | 0,000762 | 0,028344 | CDHR2 | 54825 |
| ILMN_1717888 | 1,58 | 8,76 | 0,000847 | 0,030105 | KHK | 3795 |
| ILMN_1713246 | 1,65 | 9,46 | 0,000858 | 0,030423 | SLC28A1 | 9154 |
| ILMN_2415277 | 1,86 | 7,92 | 0,000914 | 0,031591 | SLC26A9 | 115019 |
| ILMN_1725276 | -1,63 | 7,54 | 0,000927 | 0,031747 | FDCSP | 260436 |
| ILMN_1653687 | 1,88 | 9,12 | 0,000932 | 0,031885 | GALNT9 | 50614 |
| ILMN_1668052 | -1,68 | 7,55 | 0,00112 | 0,035961 | FOXA2 | 3170 |
| ILMN_1783756 | 2,93 | 8,63 | 0,001155 | 0,036583 | SLC22A8 | 9376 |
| ILMN_1795257 | 1,57 | 9,22 | 0,001252 | 0,038609 | GPT | 2875 |
| ILMN_1789648 | 1,99 | 7,77 | 0,001286 | 0,039194 | SCGN | 10590 |
| ILMN_1675947 | 2,17 | 8,64 | 0,001327 | 0,040058 | MT3 | 4504 |
| ILMN_1717565 | 1,54 | 9,18 | 0,001741 | 0,047463 | NA | 348174 |
