# Supplementary figures and images for: Patterns of gene expression characterize T1 and T3 clear cell renal cell carcinoma subtypes
Source: PLoS One. 2019 May 31;14(5):e0216793. doi: 10.1371/journal.pone.0216793 (PMC6544217; doi:10.1371/journal.pone.0216793)

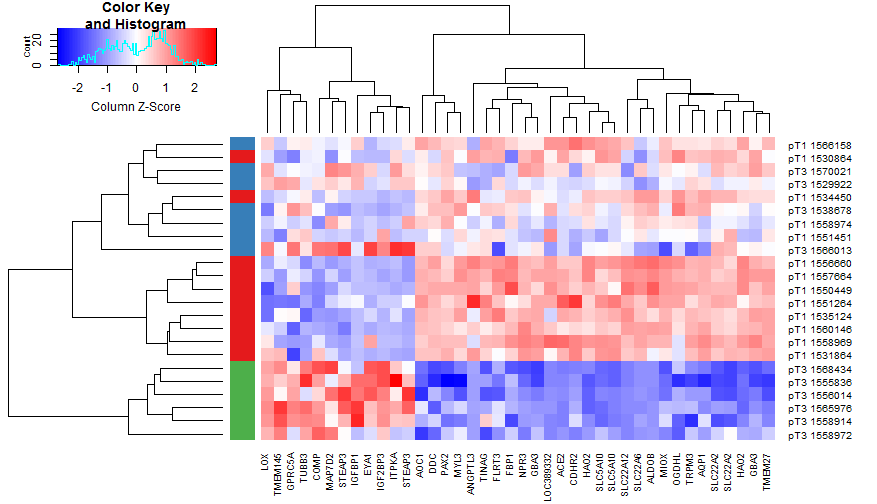

Supplement: S1 Fig — Cut-off p-value 0.05. Blue–underexpressed, red–overexpressed genes. Based on the expression pattern the samples were divided into three clusters. Colour bar indicates what cluster the sample was assigned to: red–A1 (pure T1), green–A3 (pure T3), blue–A2 (mixed). (TIFF) [file pone.0216793.s005.tiff]

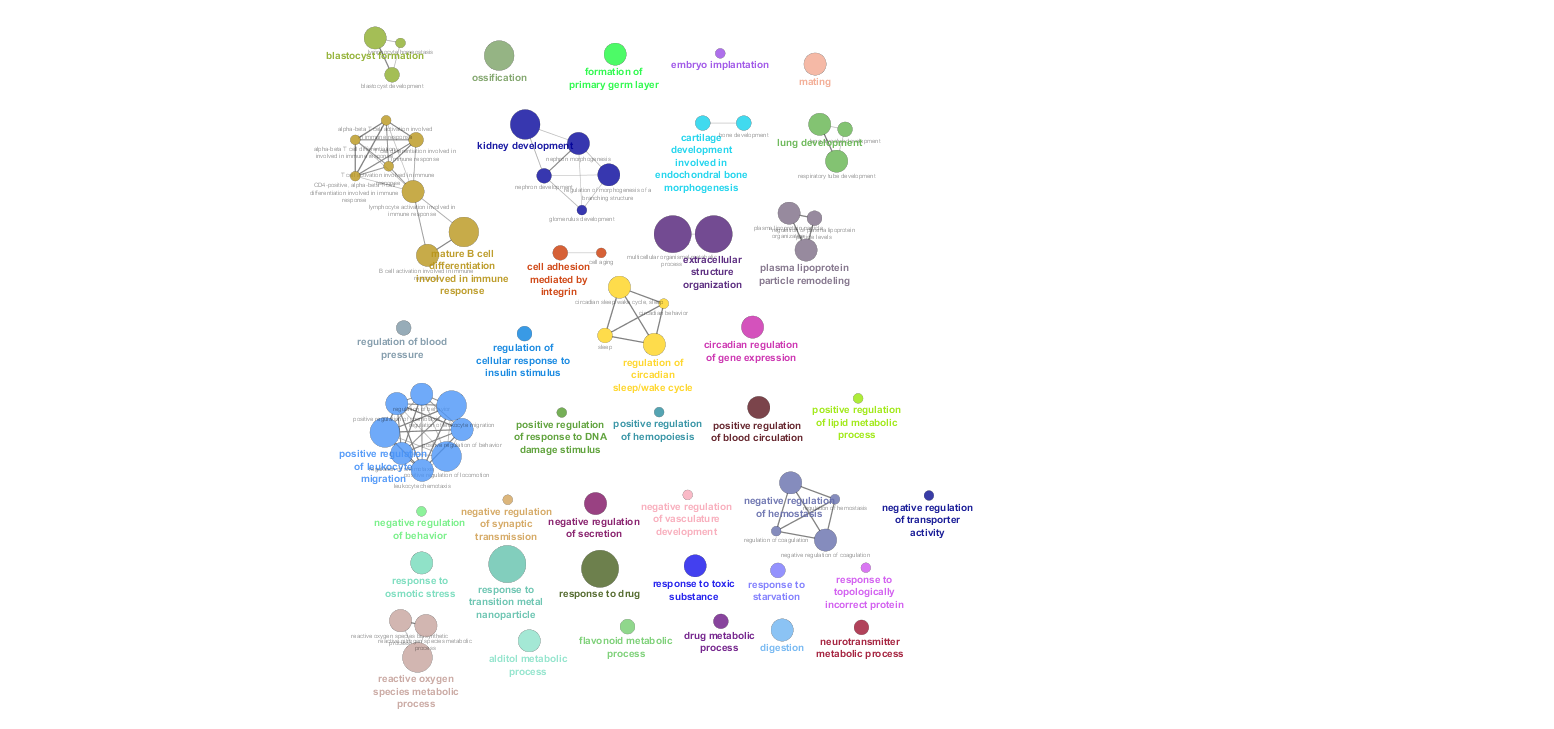

Supplement: S2 Fig — Adjusted p-value < 0.1, 481 genes. Narrowed down to genes in level 3 in the Genome Ontology (GO) hierarchy. (TIF) [file pone.0216793.s006.tif]

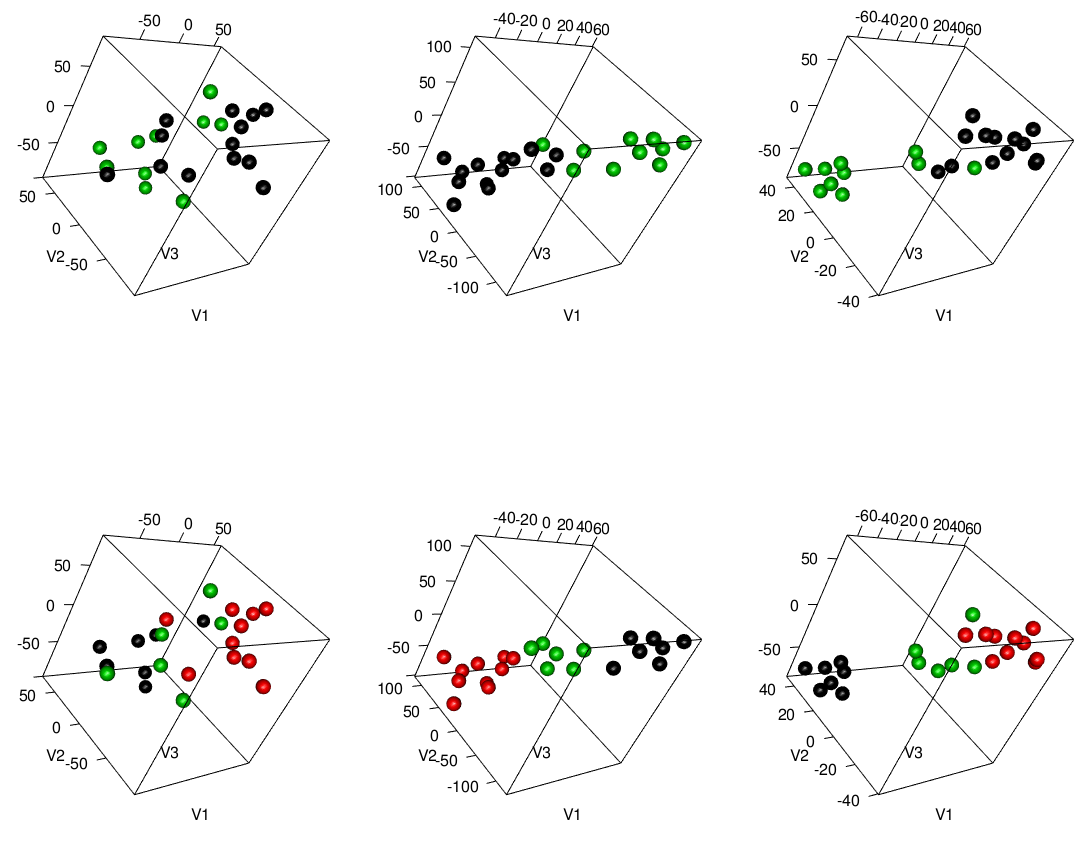

Supplement: S3 Fig — Three sets of probes were used in this analysis: (1) the probes used for hierarchical clustering (aligned to 481 genes); (2) top 40 differentially expressed probes, and (3) all 34476 probes. S were projected on a 3-dimensional space. For the unbiased case (all probes) no association between tumor size and the three components is present. Interestingly, for the two sets of pre-selected features, not only do we see a separation between T1 and T3 samples in the 3D space, but also a separation between the three clusters defined in the previous section. (TIF) [file pone.0216793.s007.tif]
